# Breast Tumor Diagnosis Based on Molecular Learning Vector Quantization Neural Networks

**DOI:** 10.1002/advs.202409150

**Published:** 2024-09-18

**Authors:** Chun Huang, Jiaying Shao, Baolei Peng, Qingshuang Guo, Panlong Li, Junwei Sun, Yanfeng Wang

**Affiliations:** ^1^ The School of Electrical and Information Engineering Zhengzhou University of Light Industry Zhengzhou Henan 450001 China

**Keywords:** breast tumor diagnosis, DNA strand displacement, learning vector quantization neural network, loser‐take‐all network, manhattan distance

## Abstract

DNA nanotechnology plays a crucial role in precise cancer medicine. Currently, molecular logic circuits are applied to detect tumor‐specific biomarkers and control the release of therapeutic drugs. However, these systems lack self‐learning capabilities for intelligent diagnostics in biological samples, and their data processing capabilities are limited. Here, a molecular learning vector quantization neural network (LVQNN) model based on DNA strand displacement (DSD) technology for breast tumor diagnosis is developed. Compared to previous work, the molecular LVQNN boasts powerful computing abilities, handling high‐dimensional data for intelligent cancer diagnosis. To verify the feasibility and versatility of the network, two distinct typical datasets are selected: one from a single source with cell morphology data from 569 cases, and a more extensive one spanning different populations and ages, with miRNA gene expression data from 1881 cases. By using the molecular LVQNN, diagnostic experiments are conducted on 50 and 120 public individuals from these two datasets, respectively, achieving accuracy rates of 94% and 97.5%. This study demonstrates that the LVQNN model exhibits significant advantages in breast cancer diagnosis and enhances diagnostic accuracy while introducing new approaches for intelligent cancer diagnosis, anticipated to bring significant breakthroughs and application prospects to precise cancer medicine.

## Graphical abstract

This study constructed a Learning Vector Quantization neural network (LVQNN) model using DNA strands, which is not constrained by input and weight values. To verify the model's stability and generalization capability, the gene expression levels of tumor‐specific miRNA markers are used as inputs for the molecular neural network, successfully enabling diagnostic experiments for female breast tumors.

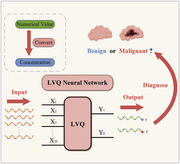

## 1 Introduction

Breast cancer has become the most common malignant tumor in women.^[^ ^1^ ^]^ Early stage of breast cancer has no obvious clinical manifestations, making it difficult to detect in time. In addition, the prevalence of this disease continues to increase and shows a trend toward younger age. Breast cancer has significant heterogeneity,^[^ ^2^ ^]^ and its pathological features include the combination of malignant cells with normal cells. Therefore, the diagnosis and treatment of breast cancer are facing huge challenges. The rapid development of nanomedicine provides strong support for the diagnosis and treatment of breast cancer. Nanoparticles that have been applied in clinical treatment, such as liposomes,^[^ ^3^, ^4^ ^]^ magnetic nanoparticles,^[^ ^5^ ^]^ and nanogels^[^ ^6^ ^]^ have achieved certain results in the diagnosis and treatment process. However, these nanomedical technologies still have deficiencies in autonomous calculation of biological samples, lacking sufficient intelligent performance.

DNA computers have attracted close attention from scientists,^[^ ^7^, ^8^, ^9^, ^10^, ^11^, ^12^ ^]^ due to their small size, large storage capacity, low energy consumption and high parallel computing capability. These computers are composed of a series of biomolecules assembled in solution and mainly rely on biochemical reactions to achieve rapid processing of molecular information. DNA strands, which are highly programmable,^[^ ^13^, ^14^, ^15^, ^16^, ^17^ ^]^ are an easily accessible nanoscale engineering material, and have been widely used in the areas of NP‐complete computational problems,^[^ ^18^ ^]^ biosensor devices,^[^ ^19^, ^20^ ^]^ nanorobotics,^[^ ^21^, ^22^, ^23^ ^]^ drug delivery,^[^ ^24^, ^25^ ^]^ disease diagnosis,^[^ ^26^, ^27^, ^28^ ^]^ and nanocircuits.^[^ ^29^ ^]^ DNA logic circuits for cancer diagnosis and treatment have been developed and have shown fewer side effects and higher therapeutic efficacy in cancer treatment.^[^ ^30^, ^31^, ^32^, ^33^, ^34^, ^35^ ^]^ However, their limited adaptability and cognitive capacity reveal the challenges they encounter when dealing with extensive and intricate tasks.

In recent years, artificial neural networks have gained increasing popularity due to their remarkable learning capabilities. Artificial neural networks have demonstrated exceptional performance in autonomously inferring complex dynamic problems. Through the process of learning and training, neural networks can efficiently extract crucial features and patterns from input data. This ability makes artificial neural networks show excellent performance in various fields such as image recognition,^[^ ^36^ ^]^ speech recognition,^[^ ^37^, ^38^ ^]^ natural language processing,^[^ ^39^ ^]^ and medical diagnosis.^[^ ^40^, ^41^ ^]^ DNA‐based artificial neural circuits have been developed. In 2011, Qian et al., from the California Institute of Technology in the United States pioneered the utilization of DNA molecules for constructing artificial neurons.^[^ ^42^ ^]^ They successfully demonstrated Hopfield associative memory and effectively stored four single‐stranded DNA patterns within this miniaturized neural network. This work combines DSD technology with neural networks, marking an important milestone in the integration of DNA biocircuits and artificial intelligence systems. Subsequently, the Qian team further optimized the DNA neural network, and established a winner‐take‐all neural network.^[^ ^43^ ^]^ The network expanded pattern recognition from 4 to 9, and successfully realized the recognition of handwritten numbers 1 to 9. This DNA neural network circuit, which is not limited by the number and complexity of patterns, provides the possibility for autonomous molecular systems to endow intelligent decision‐making power. In addition, a loser‐take‐all neural network has also been presented,^[^ ^44^ ^]^ which can identify patterns in an extremely noisy environment. Recently, Xiong et al., constructed a molecular convolutional neural network using DSD technology,^[^ ^45^ ^]^ achieving weight strand sharing and parallel convolution operations, and successfully identifying patterns of up to 32 categories. These results demonstrate that DNA neural networks have strong computing power and huge application potential, which promotes its wider application in disease diagnosis and nanomedicine.

LVQNN is an input‐forward neural network used for training the supervised learning method of the competition layer. Its algorithm is derived from Kohonen's competition algorithm and has wide applications in the field of pattern classification and optimization. Compared to other neural network algorithms, the LVQNN exhibits a concise network structure. It leverages internal unit interactions to perform highly intricate classification processing and effectively converges toward diverse scattered design conditions within the design space, thereby facilitating prompt decision‐making. Furthermore, the LVQNN eliminates the need for input vector normalization or orthogonalization by directly computing distances between the input vector and competition layer for pattern recognition, thus ensuring simplicity in operation.

In this paper, a DNA‐based LVQNN model is proposed. In LVQNN, computing the Manhattan distance is a crucial step. Manhattan distance is solved by constructing an annihilation module using DNA cooperative hybridization. What's more, a powerful loser‐take‐all network is also constructed through signal reversal and annihilation reactions, achieving the minimum distance judgment between inputs and competing layer neurons. Based on the described process, this study initially performed diagnostic experiments to assess breast tumor malignancy. Nuclear‐related data from breast tumor cells were used as input for the molecular LVQNN, validating the feasibility of the DNA‐based LVQNN. Later, experiments simulating real breast tumor diagnoses were conducted using the concentration of tumor‐specific marker miRNA strands as input. The code is designed in modules using the software Visual DSD, and simulations are performed to verify the feasibility of the designed DNA reaction networks. The DNA‐based LVQNN designed in this study overcomes the impact of leakage reactions, as detailed in Section S2 (Supporting Information). DNA neural networks have shown strong application potential in the medical field.

## 2 Results

### 2.1 DNA‐Based LVQNN

The algorithm of LVQNN belongs to the prototype clustering algorithm. Initially, a set of prototype vectors is randomly selected from the data set as cluster centers, dividing the clustering space into multiple clusters. For each input sample, it is assigned to the nearest cluster based on distance, with the requirement that the data is labeled with category tags. The core idea involves iteratively optimizing the prototype vectors corresponding to each cluster. In each iteration, for all labeled training samples, the algorithm identifies the prototype vector closest to the sample and updates it appropriately by checking whether their category labels match. The essence of the LVQNN algorithm lies in pulling closer those data points with the same label as the prototype vector while pushing away those with different labels.

**Figure 1:**
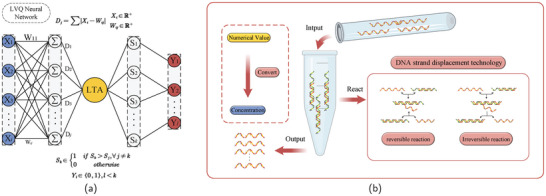
Network Model and Reaction Principles. a) DNA‐based LVQNN model. A DNA‐based LVQNN has i inputs, j competing layer neurons, and l outputs, where the number of i is independent of the number of j and the number of l is less than the number of j. *D*_*j*_ represents the distance factor, solving the distance between the input and the weight. *S*_*k*_ represents the reverse signal, j and k have the same quantity, but j ≠ k. b) Principles of DSD technology.

The LVQNN comprises three layers of neurons: input layer, competition layer, and linear output layer. As illustrated in **Figure** **1**a, there is full connectivity between the input layer and the competition layer, while partial connectivity exists between the competition layer and the linear output layer. The number of neurons in the competition layer always exceeds the number of neurons in the linear output layer. Each neuron in the competition layer is connected to only one neuron in the linear output layer, with a constant weight of 1. However, each neuron in the linear output layer can be connected to multiple neurons in the competition layer. The values of neurons in both the competition layer and the linear output layer are binary, being either “1” or “0.” When an input pattern is fed into the network, the competing layer neurons that are closest to the input pattern are activated, with its state set to “1,” while the states of other neurons in the competition layer are set to “0.” Consequently, the neurons in the linear output layer connected to the activated neuron also have a state of “1,” while the states of other neurons in the linear output layer are all “0.” The principle of DSD reaction is illustrated in Figure 1b. DSD reactions include both reversible and irreversible reactions. Using these two mechanisms, we can construct circuit modules with various functions. Here, we translate numerical quantities into concentrations of DNA strands to construct a DNA‐based LVQNN model.

**Figure 2:**
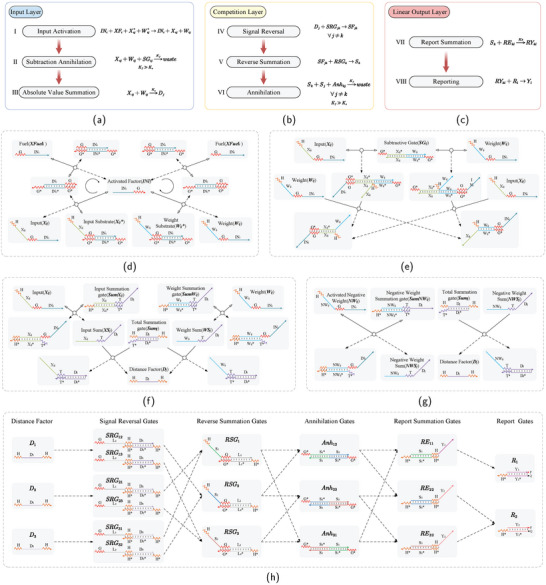
LVQNN and its DNA implementation. a) Input layer. In the reaction, waste molecules are not depicted. *K*_*f*_ represents the rate constant of the subtraction annihilation reaction, while *K*_*s*_ denotes the rate constant of the absolute value summation reaction, among *K*_*f*_ > >*K*_*s*_. b) Competitive layer. Where *K*_*f*_ is the rate constant for the annihilation reaction. c) Linear output layer. Where *K*_*s*_ is the rate constant for the reported summation reaction, and *K*_*s*_ < <*K*_*f*_. d) The DSD process in the input activation module. The gray circles with arrows denote the direction of catalytic cycles, while black and white arrows represent the forward and backward directions of reaction steps, respectively. Lines without arrows indicate irreversible reaction directions. Zigzag lines indicate short (5 or 7 nucleotides) toehold domains and straight lines indicate long (15 or 20 nucleotides) branch‐migration domains in DNA strands, with arrowheads marking their 3' ends. Each domain is labeled with a name and assigned a unique DNA sequence, with asterisks in the names indicating sequence complementarity. e) Subtraction annihilation module. f) Absolute value summation module. g) The subtraction annihilation module and absolute value summation module with negative weight substrates. h) The DSD implementation of a three‐input loser‐take‐all circuit. The reactions involve the signal reversal module, reverse summation module, annihilation module, report summation module, and reporting reaction module. The black arrow points in the direction of the forward reaction.

The DNA‐based LVQNN consists of eight modules: input activation module, subtraction annihilation module, absolute value summation module, signal reversal module, reverse summation module, annihilation module, report summation module, and reporting reaction module. The first three modules correspond to the input layer of the LVQNN, as shown in **Figure** **2**a. Modules four to six correspond to the competitive layer of the LVQNN, as depicted in Figure 2b. Modules seven and eight, as illustrated in Figure 2c, correspond to the linear output layer of the LVQNN.

The input activation module refers to the activation of inputs and weights required for a reaction. Before the introduction of the activated factor *IN*_*i*_, the concentrations of input strand *X*_*ij*_ and weight strand *W*_*ij*_ are both locked within their respective input substrates $X_{ij}^{*}$ and weight substrates $W_{ij}^{*}$. Only after the activation factor is introduced, inputs and weights are generated, proceeding to the next reaction. The subtraction annihilation module refers to the subtraction operation between input strands *X*_*ij*_ and weight strands *W*_*ij*_ through annihilation reactions. In this process, each pair of input *X*_*ij*_ and weight *W*_*ij*_ will continuously undergo annihilation until only one of the input *X*_*ij*_ or weight *W*_*ij*_ remains, and the reaction will stop. The absolute value summation module refers to the process of summing the concentrations of the remaining input strand *X*_*ij*_ or weight strand *W*_*ij*_ after the annihilation reaction has concluded. The input strands and weight strands with the same subscript “j” are summed to obtain the concentration of the distance factor *D*_*j*_. In summary, the first three modules correspond to the input layer of the LVQNN. Due to the difficulty of directly constructing a model for solving the Euclidean distance between input layer vectors and competitive layer neurons through reactions between DNA strands, we proposed an equivalent Manhattan distance solving model to address the distance between input layer vectors and competitive layer neurons.

Modules four to six construct a loser‐take‐all network to implement the competitive layer of the LVQNN. The first module is the signal reversal module, which takes the distance factor *D*_*j*_ generated from the absolute value summation module as inputs for the loser‐take‐all network. It then undergoes strand displacement reactions with the signal reversal gates *SRG*_*jk*_, distributing the concentration of the distance factor *D*_*j*_ evenly across different reversal factor *SF*_*jk*_. The reverse summation module refers to the concentration of all the reversal factor *SF*_*jk*_ with the same index “k” into a single reverse signal *S*_*k*_, while the concentration of *S*_*k*_ is the summation of all the reversal factor *SF*_*jk*_ with the same index “k.” Thus, the species with the minimum concentration in the distance factor *D*_*j*_ is reversed to the species with the maximum concentration in the reverse signal *S*_*k*_. Similarly, The magnitude to the concentrations of all species in *D*_*j*_ undergoes reversal. The next module is the annihilation module, similar to the subtraction annihilation module, where different *S*_*k*_ and *S*_*j*_ undergo pairwise annihilation reactions until only one species remains from either *S*_*j*_ or *S*_*k*_, signaling the cessation of reactions. The remaining species emerges as the winner. These three modules collectively achieve the loser‐take‐all functionality, which determines the competitive layer neuron in the LVQNN with the smallest distance from the input vector.

The final two modules correspond to the linear output layer of the LVQNN. First, the working principle of the summation reporting module is introduced as follows. The summation reporting gate *RE*_*kl*_ undergoes strand displacement reaction with the reverse signal strand *S*_*k*_, producing the reporting signal strand *RY*_*kl*_. Here, the branch migration domain *Y*_*l*_ in *RE*_*kl*_ corresponds to different linear outputs, reflecting partial connectivity patterns in the linear output layer of the LVQNN. Different branch migration domains *Y*_*l*_ combine with their respective reverse signal strands *S*_*k*_ to achieve partial connectivity functionality. Finally, the reporting reaction module achieved two objectives: first, summing the same branch migration domains *Y*_*l*_ and generating the output strand *Y*_*l*_; second, separating the fluorophore from the quencher to extract the output result of the reaction.

The input activation module is shown in Figure 2d, which is a catalytic reaction. The activated factor *IN*_*i*_, undergoes reversible strand displacement reactions with the input substrate $X_{ij}^{*}$ and the weight substrate $W_{ij}^{*}$, generating the input strand *X*_*ij*_, the weight strand *W*_*ij*_, and a common intermediate product. The intermediate product undergoes reversible strand displacement reactions once more with the fuel strand *XFuel*_*i*_, regenerating the activated factor *IN*_*i*_. Thus, as long as the activated factor *IN*_*i*_ is present and the fuel strand *XFuel*_*i*_ is sufficient, the reaction can fully convert all input substrates $X_{ij}^{*}$ and weight substrates $W_{ij}^{*}$ into their respective input strands *X*_*ij*_ and weight strands *W*_*ij*_. Here, the concentration of the activated factor *IN*_*i*_ is set equal to that of the input substrates. The subtraction annihilation module, as illustrated in Figure 2e, initiates with a reversible reaction in the first step of subtraction annihilation, where species *X*_*ij*_ or *W*_*ij*_ respectively bind to the exposed toehold in the subtractive gate *SG*_*ij*_ and migrate toward the center. The second step involves an irreversible reaction, where another DNA strand *X*_*ij*_ or *W*_*ij*_ binds to the other exposed toehold on the intermediate product strand formed in the first step and migrates toward the midpoint, ultimately disassembling the subtractive gate *SG*_*ij*_ into two waste strands. Here, the toehold utilized in the subtraction annihilation reaction is G, consisting of seven nucleotides. The absolute value summation module, as shown in Figure 2f, consists of two reactions. The first reaction involves the remaining *X*_*ij*_ or *W*_*ij*_ from the reactions of the subtraction annihilation module undergoing reversible strand displacement reactions with the Input Summation gate *SumX*_*ij*_ or the Weight Summation gate *SumW*_*ij*_ respectively. This generates the Input Sum strand *XS*_*j*_ and the Weight Sum strand *WS*_*j*_. The second reaction involves the Input Summation gate *XS*_*j*_ and the Weight Summation gate *WS*_*j*_ respectively undergoing irreversible strand displacement reactions with the Total Summation gate *Sum*_*j*_, generating the distance factor *D*_*j*_. In the resulting double‐stranded products of this reaction, exposed toeholds are no longer present, rendering the reaction irreversible. The irreversible reaction drives the first reversible reaction forward until all input strands *X*_*ij*_ and weight strands *W*_*ij*_ have been fully reacted. As shown in Figure 2g, when the weight is negative, the branch migration domain *W*_*ij*_ is converted to *NW*_*ij*_. In this case, the branch migration domain *NW*_*ij*_ of the activated negative weight strand will no longer match the branch migration domain *W*_*ij*_ of the subtraction gate, resulting in the DNA strand representing the negative weight, *NW*_*ij*_, no longer participating in the subtraction annihilation reaction. The corresponding input strand *X*_*ij*_, when engaged in the subtraction annihilation reaction, undergoes only the reversible first part of the reaction due to having only one reactant, thus remaining entirely unconsumed. The first part of the reaction in the absolute value summation module utilizes the foothold H, which is present on both the input strand *X*_*ij*_ and the weight strand *W*_*ij*_. This foothold contains five nucleotides. It is well‐known that the rate of strand displacement reactions exponentially increases with the length of the toehold. Previous studies have indicated that the difference in reaction rates between toeholds of four nucleotides and seven nucleotides can reach astonishing magnitudes, exceeding thousands of times.^[^ ^46^, ^47^ ^]^ Here, the difference in rates between toeholds G and H is on the order of hundreds, resulting in a reaction rate of the absolute value summation module much slower than that of the subtraction annihilation reaction. The second step of the absolute value summation reaction utilizes the toehold T, which also contains five nucleotides. The reason for changing the toehold here is to reduce the formation of unnecessary polymers.

The reactants and products of the signal reversal module, the reverse summation module, the annihilation module, the report summation module, and the reporting module are illustrated in Figure 2h. Both the signal reversal module and the reverse summation module are irreversible DNA strand replacement reactions, and the double‐stranded products of the reaction no longer contain the exposed foothold, and the products of the previous reaction are the reactants of the next reaction, and all the reactions occur naturally in sequence. The annihilation reaction module is similar to the subtraction annihilation reaction module. A reverse signal strand *S*_*k*_ can bind to one side of the toehold on the annihilation chain molecule *Anh*_*kj*_ and migrate to the midpoint of the double‐stranded domain. If only species *S*_*k*_ is present, this process is entirely reversible, and no molecules are consumed. However, if another reverse signal strand *S*_*j*_ is also present, it can bind to the opposite toehold on the annihilation chain and migrate to the midpoint of the double‐stranded domain. When both *S*_*k*_ and *S*_*j*_ strands simultaneously reach the midpoint, the annihilation chain *Anh*_*kj*_ will split into two waste molecules. Since both waste molecules lack exposed toeholds, they cannot interact with any other molecules. Here, the toehold utilized in the annihilation reaction is G, consisting of seven nucleotides. The strand displacement reaction of the report summation module is reversible, with the reaction proceeding in the direction of consuming the report signal strand *RY*_*kl*_. The toehold utilized in the report summation reaction is H, consisting of five nucleotides. The reaction of the summation reporting module is an irreversible strand displacement reaction, where the product of the preceding reaction serves as the reactant for the subsequent reaction, driving the reaction forward in the positive direction. In the Supporting Information section, specifically in Chapter S1 (Supporting Information), as illustrated in Figures S1 through S11 (Supporting Information), we have detailed the chemical reaction networks associated with each individual module. These figures provide a comprehensive depiction of the intricate interactions and transformations that occur within each module, serving as a fundamental basis for understanding the overall functionality and performance of thesystem.

### 2.2 Implement of the Input Layer in LVQNN

**Figure 3:**
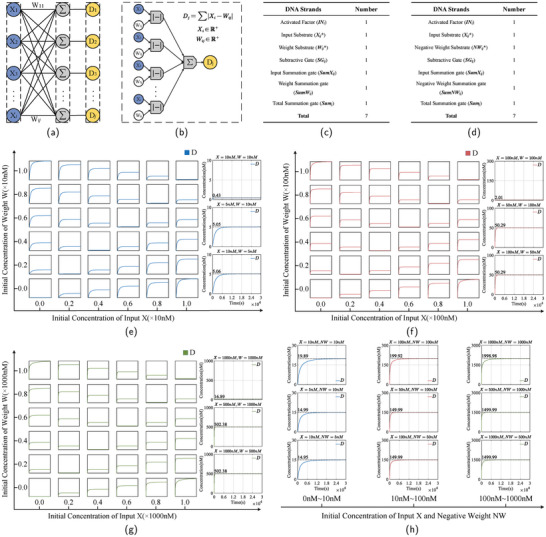
Solving Manhattan distance. a) DNA‐based LVQNN input layer model. b) Flow chart of the subtraction annihilation module and the absolute value summation module. c) Table of the number of DNA species required for reactions when weights are positive. d) Table of the number of DNA species required for reactions when weights are negative. e) Behavior of subtraction annihilation and absolute value summation for different combinations of input *X* and weight *W* within the 0–10 nM input range. *D* represents the distance factor generated by the absolute value summation reaction. In the reactions, the concentrations of subtraction gate *SG*_*ij*_, input summation gate *SumX*_*ij*_, weight summation gate *SumW*_*ij*_, and total summation gate *Sum*_*j*_ are all set to 12 nM. Each subplot illustrates the relationship between normalized signal (ranging from 0 to 1 on the x‐axis) and time (ranging from 0 to 30 000 s on the y‐axis). f) Behavior of subtraction annihilation and absolute value summation for different combinations of input *X* and weight *W* within the 0–100 nM input range. In the reactions, the concentrations of subtraction gate *SG*_*ij*_, input summation gate *SumX*_*ij*_, weight summation gate *SumW*_*ij*_, and total summation gate *Sum*_*j*_ are all set to 120 nM. g) Behavior of subtraction annihilation and absolute value summation for different combinations of input *X* and weight *W* within the 0–1000 nM input range. In the reactions, the concentrations of subtraction gate *SG*_*ij*_, input summation gate *SumX*_*ij*_, weight summation gate *SumW*_*ij*_, and total summation gate *Sum*_*j*_ are all set to 1200 nM. h) When the weights are negative, the behavior of subtraction annihilation and absolute value summation for different combinations of input *X* and weight *W* within the input ranges of 0–10, 10–100, and 100–1000 nM.

The input layer of the LVQNN requires calculating the distance between input vectors and neurons in the competitive layer. In this paper, a DNA strand‐based Manhattan distance computation model is proposed to address the distance computation problem. **Figure** **3**a illustrates the input layer model of the DNA‐based LVQNN, while Figure 3b depicts the schematic of the subtraction annihilation and absolute value summation. In order to verify the feasibility of Manhattan distance calculation by subtraction annihilation module and absolute sum module, different levels of input concentration and weight concentration are designed for testing. The testing is divided into two scenarios: when the weight is positive and when the weight is negative. Since the input data contained quantity levels ranging from 0 to 1000 nM, three concentration levels were selected for each scenario: 0–10, 0–100, and 0–1000 nM. For each concentration level, representative data are chosen for testing, including the following three cases: the concentration of input *X* equals the concentration of weight *W*, the concentration of input *X* is less than the concentration of weight *W*, and the concentration of input *X* is greater than the concentration of weight *W*. The reaction results are all under ideal conditions.

When the weights are positive, the number of DNA species required for the reaction is depicted in Figure 3c. The reaction outcomes within the concentration range of 0–10 nM are illustrated in Figure 3e. The error is 4.30% when the concentration of the input strand *X* equals that of the weight strand *W*. The error is 1.00% when the concentration of *X* is less than that of *W*, and the error is 1.20% when the concentration of *X* exceeds that of *W*. The reaction outcomes within the concentration range of 10–100 nM are depicted in Figure 3f. When the concentration of the input strand *X* equals that of the weight strand *W*, the error is 2.01%; when the concentration of *X* is less than that of *W*, the error is 0.58%; and when the concentration of *X* exceeds that of *W*, the error is 0.58%. The reaction outcomes within the concentration range of 100–1000 nM are illustrated in Figure 3g. When the concentration of *X* equals that of *W*, the error is 1.69%; when the concentration of *X* is less than that of *W*, the error is 0.48%; and when the concentration of *X* exceeds that of *W*, the error is 0.48%. In summary, when the weights are positive, the subtraction annihilation module performs best within the concentration range of 100–1000 nM, followed by the range of 10–100 nM, and finally the range of 0–10 nM. However, even in the lowest concentration range, the maximum error is only 4.30%. This error corresponds to just 0.43 nM within the maximum concentration range of 10 nM, which generally does not significantly affect the reaction outcomes when dealing with most data. We observed that when the concentration of the input strand *X* equals the concentration of the weight strand *W*, the reaction errors are relatively high across all three concentration ranges. This is because when the concentration of *X* matches that of *W*, there are no surplus input strands *X* or weight strands *W* available to drive the reaction toward the decomposition and annihilation of the strands. Consequently, the reaction errors are relatively large. However, within the same low concentration range, the errors of all generated products are proportionally equivalent, thus not affecting subsequent reaction outcomes.

When the weights are negative, the required number of DNA species for the reaction is illustrated in Figure 3d, and the reaction outcomes are depicted in Figure 3(h). Within the concentration range of 0–10 nM, when the concentration of the input strand *X* exceeds that of the negative weight strand *NW*, the error is ≈0.55%; when the concentration of *X* is less than that of *NW*, the error is ≈0.06%; and when the concentration of *X* equals that of *NW*, the error is 0.33%. Within the range of 10–100 nM, when the concentration of *X* exceeds that of *NW*, the error is ≈0.04%; when the concentration of *X* is less than that of *NW*, the error is ≈0.01%; and when the concentration of *X* equals that of *NW*, the error is 0.01%. Within the range of 100–1000 nM, when the concentration of *X* exceeds that of *NW*, the error is 0.05%; when the concentration of *X* is less than that of *NW*, the error is 0.00%; and when the concentration of *X* equals that of *NW*, the error is 0.00%. In conclusion, when the weights are negative, the reaction performs excellently across all three concentration ranges, with the maximum error being only 0.55%. Therefore, our designed subtraction annihilation module and absolute value summation module are entirely feasible and can successfully compute the Manhattan distance between the input vector and the competitive layer neurons.

### 2.3 Implement of the Competitive Layer in LVQNN

**Figure 4:**
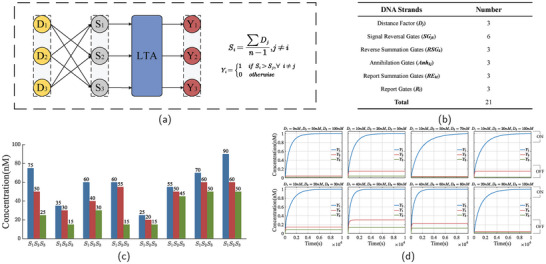
Determination of minimum distance. a) Model of the loser‐take‐all network. The loser‐take‐all network is built upon the winner‐take‐all network, achieving a simple and powerful loser‐take‐all network by utilizing the signal reversal strands *SRG*_*jk*_ and annihilation strands *Anh*_*kj*_. b) Table showing the required number of DNA species for reactions. c) The concentration of the reverse signal *S*_*k*_ under eight different input combinations. (The figure shows the concentration profile at the X‐axis coordinate time = 10 000 s.) d) Fluorescence kinetics data of three‐input (total:eight input combinations) loser‐take‐all network. All data are individually normalized, with signal reversal gate *SRG*_*jk*_ concentration set to 100 nM, reverse summation gate *RSG*_*k*_ concentration set to 100 nM, annihilation strand *Anh*_*kj*_ concentration set to 108 nM, report summation gate *RE*_*kl*_ concentration set to 100 nM, and report gate *R*_*l*_ concentration set to 150 nM.

The competitive layer of an LVQNN requires selecting the neuron with the minimum distance to the input vector. In this paper, we achieve this by constructing a DNA “loser‐take‐all” network. “Loser‐take‐all” means that the output signal is “1” only when the corresponding input has the minimum simulated value among all inputs. **Figure** **4**a illustrates the model of the DNA loser‐take‐all reaction network. Here, we demonstrate a loser‐take‐all network with a three‐input configuration, where the required number of DNA species for the reaction is depicted in Figure 4b. The fluorescence kinetics data of the loser‐take‐all network is shown in Figure 4c, where eight common input combinations are listed to evaluate the computational capability of the loser‐take‐all network. Due to the identical characteristics among the three inputs, we designate *D*_1_ as the minimum input signal for experimentation. As evident from the graph, the loser‐take‐all network successfully identifies the signal with the lowest concentration and outputs “1.”The competition layer of LVQNN needs to select the neuron with the smallest distance from the input vector. To achieve this goal, a DNA loser‐take‐all network is constructed in this paper. “Loser‐take‐all” means that the output signal is “1” only when the corresponding input has the minimum simulated value among all inputs. Figure 4a illustrates the model of the DNA loser‐take‐all reaction network. Here, we demonstrate a loser‐take‐all network with three inputs, where the required number of DNA species for the reaction is depicted in Figure 4b. The fluorescence kinetics data of the loser‐take‐all network is shown in Figure 4d, where eight input combinations are listed to evaluate the computational capability of the loser‐take‐all network. Due to the identical characteristics among the three inputs, we designate *D*_1_ as the minimum input signal for experimentation. As evident from the figure, the loser‐take‐all network successfully identifies the signal with the lowest concentration and outputs “1”. As shown in Figure 4c, the concentrations of the reverse factor *S*_*k*_ under eight different inputs all exhibit a reversal. The order of the concentrations originally was *D*_1_ < *D*_2_ < *D*_3_, but after reversal, it changed to *S*_1_ > *S*_2_ > *S*_3_.

From the fluorescence kinetics data of the reaction, we observe residual species apart from the target output. This primarily occurs due to two reasons. First, in a loser‐take‐all circuit with n inputs, any differences between reverse signals are diminished to 1/(*n* − 1) of the differences between the original inputs. For a three‐input circuit, this is expected to reduce by 50%, making competition more challenging. Another significant factor is that the reporting reaction shares reactants with the annihilation reaction. Before the reverse signal *S*_*k*_ encounters the annihilation strand, part of the reverse signal *S*_*k*_ interacts with the summation reporting gate *RE*_*kl*_. The higher the remaining concentration of the *S*_*k*_ species, the greater its chance of escaping complete extinction. These observations suggest that DNA circuits do not exhibit perfect loser‐take‐all behavior. However, for competitors that are not very similar or strong, its computation is accurate. In summary, the loser‐take‐all circuit designed in this paper is entirely feasible and can accurately distinguish the true winner when faced with certain concentration differences in the distance factors *D*_*j*_.

### 2.4 Feasibility Verification of LVQNN Based on DNA

**Figure 5:**
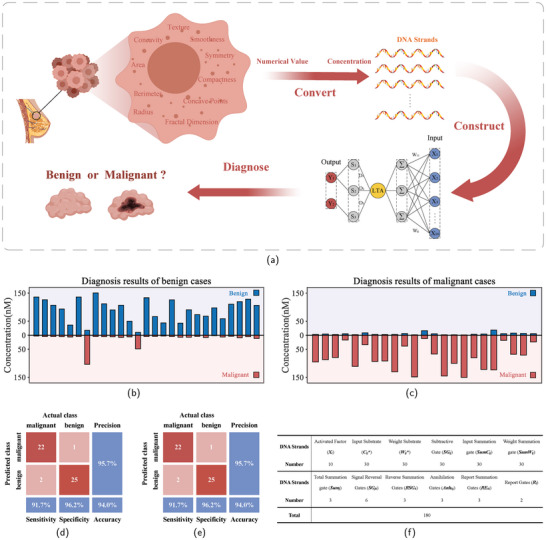
Diagnostic model and results of breast tumors. a) Flowchart of DNA‐based LVQNN for the diagnosis of breast tumor response. b)Results for diagnosing benign cases. The figure shows the concentration profile at the X‐axis coordinate time = 30 000 s. (The complete process of the reaction is shown in Figure S22, Supporting Information). c) Results for diagnosing malignant cases (complete process of the reaction is shown in Figure S23, Supporting Information). d) Analysis of the Confusion Matrix Based on Silicon‐Based Computer Diagnostic Results. e) Analysis of the Confusion Matrix for Diagnostic Results Based on DNA‐Based LVQNN. f) Table of the number of DNA strands required for the response.

After outlining the construction principles of the DNA‐based LVQNN, we validated the network's feasibility through a case study. According to medical research, distinct data disparities exist between the lesion cells of breast tumors and normal tissue cells, which can serve as a foundation for discerning benign and malignant breast tumors. There are ten quantified features coming from breast tumor cell nuclei, which are the radius, texture, perimeter, area, smoothness, compactness, concavity, concave points, symmetry, and fractal dimension. Here, data were collected from the Wisconsin Breast Cancer data set on 569 patients with breast tumors, including 357 benign tumors and 212 malignant tumors. Each case's data set consists of ten feature quantities per nucleus in the sampled tissue, including the mean, standard deviation, and worst‐case value (the average of the three largest data points for each feature), resulting in a total of thirty data points. In the data file, each set of data consists of 32 fields. The first field represents the case ID, the second field indicates the diagnosis result (B for benign, M for malignant), and fields 3 to 12 represent the mean values of the 10 quantified features of cell nuclei in the tumor lesion tissue for that case. Fields 13 to 22 represent the corresponding standard deviations, and fields 23 to 32 represent the corresponding worst values. For experimentation purposes, we will select the data from fields 1 to 12, which include the case ID, diagnosis result, and the mean values of the 10 features of cell nuclei for each case. As depicted in **Figure** **5**a, the mean values of the 10 quantified features of cell nuclei in breast tumor lesion tissue serve as input *IN*_*i*_ to the network. The diagnostic results of breast tumor, denoted as *Y*_*l*_, serve as the network's output, where *Y*_*l*_ takes values of 1 or 0. Definition: When *Y*_1_ = 1, the breast tumor is diagnosed as benign nature, and then, *Y*_2_ = 0; When *Y*_2_ = 1, the breast tumor is diagnosed as malignant nature, and then, *Y*_1_ = 0. The study randomly selected 519 sets of data as the training set, with the remaining 50 sets designated as the test set. The designed LVQNN was trained using the training set data, followed by testing the test set data and analyzing the test results. Here, weight training was accomplished by silicon‐based computers. The optimal number of neurons was determined to be 3 through K‐fold cross‐validation. At this configuration, the LVQNN demonstrated the fastest training speed and the highest accuracy in diagnosing breast tumors. Therefore, an LVQNN model was constructed utilizing three competitive layer neurons. After iterations on silicon‐based computers, the LVQNN obtained results for 50 data sets in the test set, comprising 27 cases of benign tumors and 23 cases of malignant tumors.

Figure 5b illustrates the diagnostic results for benign cases, while Figure 5c illustrates the diagnostic results for malignant cases. As expected, the DNA‐based LVQNN successfully conducted diagnoses for both benign and malignant breast tumor cases. In the figure, the outputs for benign diagnoses are shown on the positive half‐axis, while the outputs for malignant diagnoses are displayed on the negative half‐axis. Among them, 25 benign diagnoses were correct, and 22 malignant diagnoses were correct.

Figure 5d shows the confusion matrix for the diagnostic results of the Silicon‐Based Computer, the silicon‐based computer correctly identified 25 out of 27 benign cases and misdiagnosed 1 out of 23 malignant cases. The overall accuracy reached 94%. Figure 5e shows the confusion matrix for the diagnostic results of the DNA‐based LVQNN. From this figure, it is evident that the molecular neural network constructed in this study achieved the same accuracy as the computer in diagnosing the malignancy and benignity of breast cancer. This indicates that our molecular neural network perfectly replicated the diagnostic results of the silicon‐based computer. These results affirm the feasibility of our designed DNA‐based LVQNN, demonstrating its capability to execute complex predictive functions. The number of DNA species required for the reaction is illustrated in Figure 5f. The DNA based LVQNN model proposed in this study only needs 180 DNA strands to accomplish complex breast cancer diagnosis tasks.

### 2.5 Breast Tumour Diagnosis with DNA‐Based LVQNN

**Figure 6:**
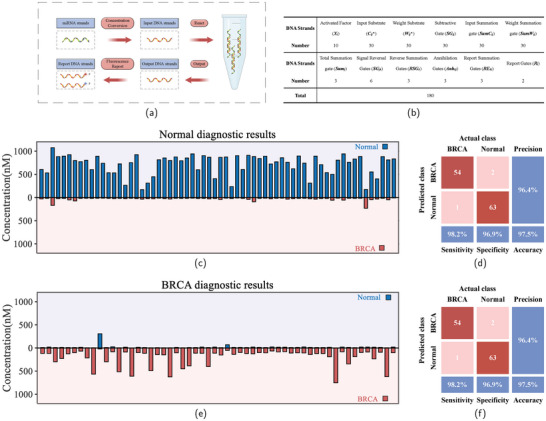
Simulated diagnostic experiments for real breast tumors. a) Flowchart for diagnosing breast tumors. b) Table of DNA strands required for the reaction. c) Diagnostic results for healthy cases. The figure shows the concentration distribution at the X‐axis coordinate time = 30 000 s (The complete process of the reaction is shown in Figure S23, Supporting Information). d) Analysis of the confusion matrix based on the silicon‐based computer diagnostic results. e) Diagnostic results for BRCA cases (The complete process of the reaction is shown in Figure S24, Supporting Information). f) Analysis of the confusion matrix for diagnostic results based on the DNA‐based LVQNN.

Next, we simulated breast tumor diagnostic experiments using the DNA‐based LVQNN developed in this study. Research indicates that serum levels of various microRNAs can serve as biomarkers for cancer diagnosis. We obtained publicly available breast miRNA data from The Cancer Genome Atlas Program (TCGA) for 1006 breast invasive carcinoma (BRCA) patients and from the cBioPortal database for 875 healthy individuals. The dataset included individuals of various ages and backgrounds. The data were cleaned and processed to remove duplicates and missing values. We performed differential expression analysis using a LASSO regression model, ranking the miRNAs by their impact factors. Based on these impact factors and data characteristics, we selected the gene expression levels of 10 miRNAs as input for the molecular LVQNN. From the 1881 publicly available datasets, we selected 1761 for the training set and 120 for the test set. The test set comprised 56 BRCA samples and 64 healthy samples. As shown in **Figure** **6**a, we converted the miRNA concentrations to equivalent DNA strand concentrations for input and conducted the diagnostic experiments.

Figure 6d,f shows the confusion matrix analysis of the computer diagnostic results and the molecular LVQNN diagnostic results, respectively, both of which remain consistent. Figure 6c shows that the diagnostic results for healthy cases correctly identified 63 cases and misdiagnosed one case, yielding a sensitivity of 96.9%. Figure 6e presents the diagnostic results for BRCA cases, correctly identifying 54 cases and misdiagnosing two cases, with a sensitivity of 98.2%. The molecular LVQNN achieved a specificity of 96.4% and an overall accuracy of 97.5% for diagnosing BRCA. This demonstrates that the molecular neural network developed in this study has strong stability and accuracy. Figure 5b illustrates the number of different types of DNA strands required for the reaction. The DNA‐based LVQNN model proposed in this study requires just 180 DNA strands to perform the complex breast cancer diagnostic task.

In summary, our DNA‐based LVQNN model exhibited excellent performance in classification tasks across two different datasets. The first diagnostic case used a smaller dataset from a single source, representing macroscopic cell morphology data. The second diagnostic case used a larger dataset that included data from various populations and age groups, representing microscopic gene expression data. Despite the significant differences in dataset size and characteristics, our LVQNN model achieved classification accuracies of 94% and 97.5%, respectively, on these datasets. By using these two different data types, diagnoses can be made at both macroscopic and microscopic levels, offering more comprehensive diagnostic information. This result indicates that our molecular neural network possesses strong generalization capabilities. However, the construction and application of molecular neural networks require further research. Future work should focus on optimizing the autonomous adjustment mechanisms for molecular network weights and handling classification tasks with large‐scale output targets. Further efforts are needed to advance molecular neural networks from research simulations to clinical practice.

## 3 Conclusion

The DNA‐based LVQNN designed in this study avoids the multiplication computation between inputs and weights at the input layer. Consequently, inputs and weights are not constrained by numerical magnitudes, and further improving the computing capacity of the neural network. The diagnostic experiments on female breast tumors showcase the robust performance of the DNA‐based LVQNN. To solve the Manhattan distance between input vectors and neurons in the competitive layer, we employed a molecular architecture based on DNA cooperative hybridization. This simple and powerful structure endows DNA molecules with flexible computational capabilities. We also devised an improved loser‐take‐all architecture to achieve the minimal distance between input vectors and neurons in the competitive layer. The research results of this article indicate that the computational prowess of DNA‐based LVQNN surpasses that of traditional DNA logic circuits, showcasing an intelligence embedded within molecular circuits. Such behavior of embedding cognitive abilities in molecular circuits opens new avenues for the advancement of molecular medicine. In future applications, the network performance can be further optimized to achieve precise detection of cancer stages and types. Moreover, in vivo cancer diagnosis can be facilitated by transcribing tumor‐specific marker miRNAs into single‐stranded DNA, which can then serve as inputs for a DNA‐based LVQNN (Learning Vector Quantization Neural Network) system. This approach has the potential to enable real‐time and accurate diagnosis of cancer within the biological environment, thereby advancing personalized medicine and early intervention strategies.

## 4 Experimental Section

This study utilizes the Visual DSD software for simulations, defining the environment as a deterministic simulation, and configuring the settings to allow polymer production with a default leak rate of 1*M* ^−1^ *s* ^−1^. The binding rate for small fulcrum G was defined as 9 × 10^7^ *M* ^−1^ *s* ^−1^, with an unbinding rate of 0.1*s* ^−1^. The binding rate for small fulcrum H was defined as 3 × 10^5^ *M* ^−1^ *s* ^−1^, with an unbinding rate of 26*s* ^−1^. The binding rate for small fulcrum T was defined as 3 × 10^5^ *M* ^−1^ *s* ^−1^, with an unbinding rate of 26*s* ^−1^. In Figures 4c, the normalization shown is performed individually, retaining two decimal places. The formula used is:

(1) $$Normalizedvalue=\frac{value-Minvalue}{Maxvalue-Minvalue}$$

The concentration of the activated factor *IN*_*i*_ and the input substrate $X_{ij}^{*}$ was set to the average value of ten quantified characteristics of tumor cell nuclei, representing as *a*_*i*_ nM. The concentration of the weight substrate $W_{ij}^{*}$ utilizes the weight values trained on the silicon‐based computer, denoting as *b*_*ij*_ nM. The concentration of the fuel chain *XFuel*_*i*_ was set to [$200\%\times (a_{i}+b_{ij})$] nM; the concentration of the subtractive gate *SG*_*ij*_ was set to [$120\%\times min(a_{i},b_{ij})$] nM; the concentrations of the input summation gate *SumX*_*ij*_ and the weight summation gate *SumW*_*ij*_ were both set to [$120\%\times |(a_{i}-b_{ij})|$] nM. The concentration of the total summation gate *Sum*_*j*_ was set to [$120\%\times \sum_{i=1}^{10}\left|a_{i}-b_{ij}\right|$] nM. Assume the concentration of the distance factor was *d*_*j*_ nM. The concentration of all signal reversal gates *SRG*_*jk*_ was set to [$100\%\times max(d_{j})$] nM. The concentration of the reverse summation gate *RSG*_*k*_ was set to [$100\%\times max(d_{j})$] nM. Assume the concentration of the reverse signal was *s*_*k*_ nM. The concentration of the annihilation gate *Anh*_*kj*_ was set to [$120\%\times max(s_{k})$] nM. The concentration of the report summation gate *RE*_*kl*_ was set to [$100\%\times max(s_{k})$] nM. The concentration of the report gate *R*_*l*_ was set to [$150\%\times max(s_{k})$] nM.

## Conflict of Interest

The authors declare no conflict of interest.

## Supporting information

Supporting Information

## Data Availability

The data that support the findings of this study are available from the corresponding author upon reasonable request.
