# Supplementary material for: Breast Tumor Diagnosis Based on Molecular Learning Vector Quantization Neural Networks
Source: Adv Sci (Weinh). 2024 Sep 18;11(42):2409150. doi: 10.1002/advs.202409150 (PMC11558146; doi:10.1002/advs.202409150)
Supplement: Supplementary file 1 — Supporting Information [file ADVS-11-2409150-s001.pdf]

ADVANCED SCIENCE
Open Access

Supporting Information

for *Adv. Sci.*, DOI 10.1002/advs.202409150

Breast Tumor Diagnosis Based on Molecular Learning Vector Quantization Neural Networks

*Chun Huang, Jiaying Shao, Baolei Peng, Qingshuang Guo, Panlong Li, Junwei Sun and Yanfeng Wang**

Breast tumor diagnosis based on molecular learning vector quantization neural networks

Support Information

*Chun Huang Jiaying Shao Baolei Peng Qingshuang Guo Panlong Li Junwei Sun Yanfeng Wang**

C. Huang, J. Shao, B. Peng, Q. Guo, P. Li, J. Sun, Y, Wang
Address: The School of Electrical and Information Engineering, Zhengzhou University of Light Industry
Email Address: yanfengwang@yeah.net;

S1 Modeling and Simulation

In order to better understand the reaction process of the DNA-based LVQNN constructed in this article, a Chemical Reactor Network (CRN) model was constructed for each module to analyze the reaction process. Among them, IP represents intermediate products, waste represents waste chain, and there is no exposed foothold in the waste chain, so it cannot interact with any other molecules.

CRN	The process of reaction
$X^*_{ij} + IN_i \underset{k_{rf}}{\overset{k_f}{\rightleftharpoons}} X^*_{ij}{:}IN_i$	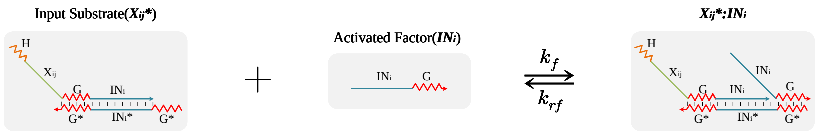
$X^*_{ij}{:}IN_i \underset{k_f}{\overset{k_{rf}}{\rightleftharpoons}} IP_1 + X_{ij}$	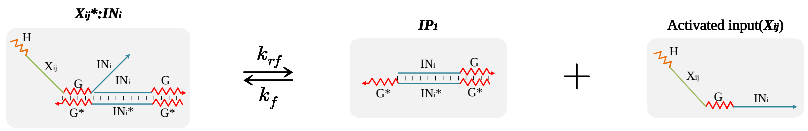
$W^*_{ij} + IN_i \underset{k_{rf}}{\overset{k_f}{\rightleftharpoons}} W^*_{ij}{:}IN_i$	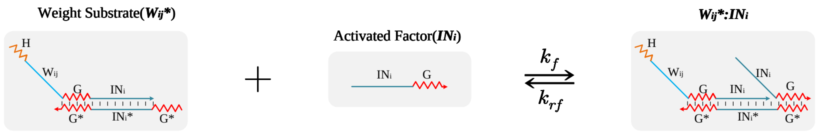
$W^*_{ij}{:}IN_i \underset{k_f}{\overset{k_{rf}}{\rightleftharpoons}} IP_1 + W_{ij}$	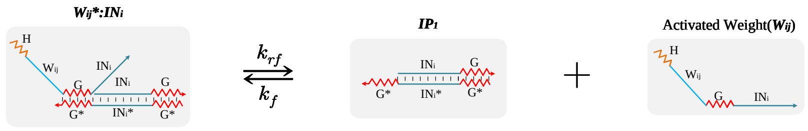
$IP_1 + XF_i \underset{k_{rf}}{\overset{k_f}{\rightleftharpoons}} IP_1{:}XF_i$	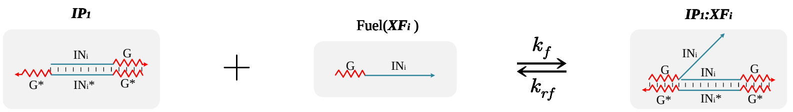
$IP_1{:}XF_i \underset{k_f}{\overset{k_{rf}}{\rightleftharpoons}} IP_2 + IN_i$	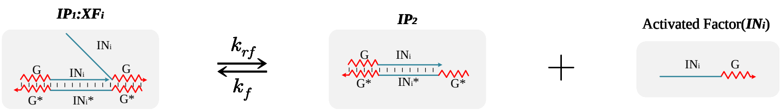

Figure S1: CRN model and reaction process of the input activation module, where $k_f = 9 \times 10^7 M^{-1}s^{-1}$, $k_{rf} = 0.1s^{-1}$.

CRN	The process of reaction
$SG_{ij} + X_{ij} \underset{k_{rf}}{\overset{k_f}{\rightleftarrows}} SG_{ij}{:}X_{ij}$	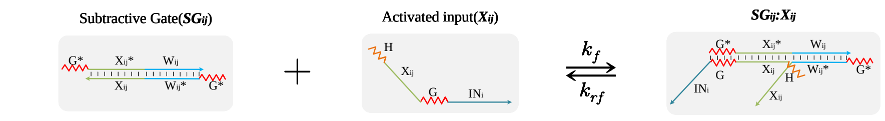
$SG_{ij}{:}X_{ij} + W_{ij} \xrightarrow{k_f} waste1 + waste2$	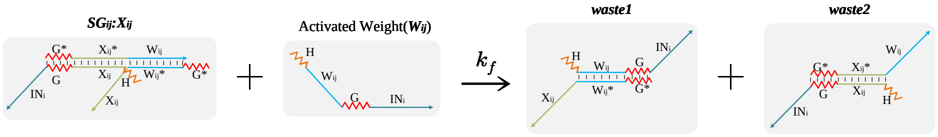
$SG_{ij} + W_{ij} \underset{k_{rf}}{\overset{k_f}{\rightleftarrows}} SG_{ij}{:}W_{ij}$	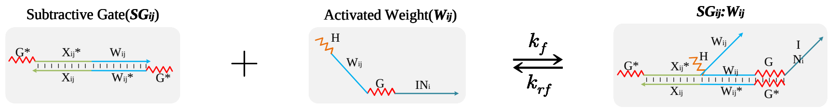
$SG_{ij}{:}W_{ij} + X_{ij} \xrightarrow{k_f} waste1 + waste2$	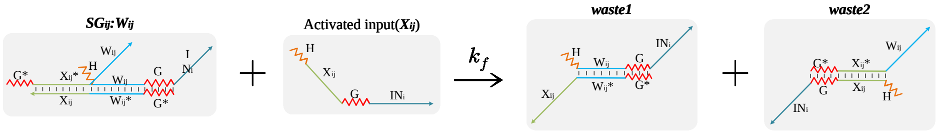

Figure S2: CRN model and reaction process of the subtractive annihilation module, where $k_f = 9\times10^7 M^{-1}s^{-1}$, $k_{rf} = 0.1s^{-1}$.

CRN	The process of reaction
$SG_{ij} + X_{ij} \underset{k_{rf}}{\overset{k_f}{\rightleftarrows}} SG_{ij}{:}X_{ij}$	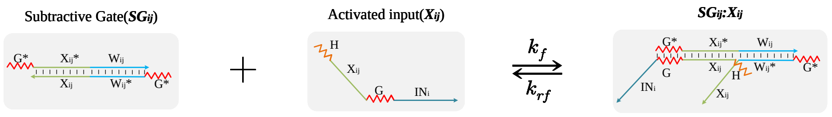

Figure S3: CRN model and reaction process of the subtractive annihilation module when the weight is negative, where $k_f = 9 \times 10^7 M^{-1}s^{-1}$, $k_{rf} = 0.1s^{-1}$.

CRN	The process of reaction
$SumX_{ij} + X_{ij} \underset{k_{rs}}{\overset{k_s}{\rightleftharpoons}} SumX_{ij}{:}X_{ij}$	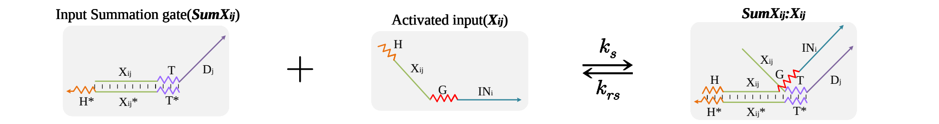
$SumX_{ij}{:}X_{ij} \underset{k_s}{\overset{k_{rs}}{\rightleftharpoons}} IP_3 + XS_{ij}$	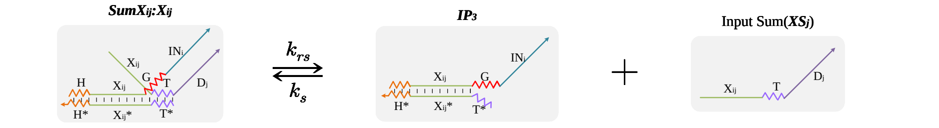
$SumW_{ij} + W_{ij} \underset{k_{rs}}{\overset{k_s}{\rightleftharpoons}} SumW_{ij}{:}W_{ij}$	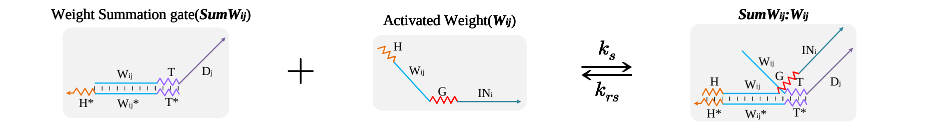
$SumW_{ij}{:}W_{ij} \underset{k_s}{\overset{k_{rs}}{\rightleftharpoons}} IP_4 + WS_{ij}$	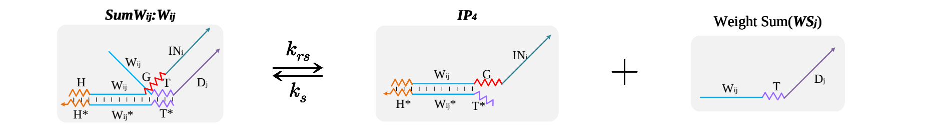
$Sum_j + XS_{ij} \xrightarrow{k_s} waste3 + D_j$	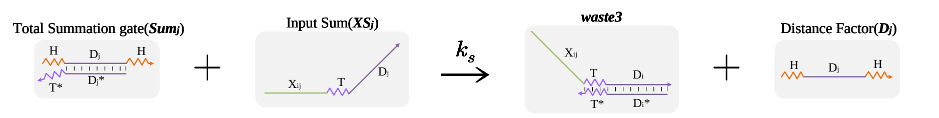
$Sum_j + WS_{ij} \xrightarrow{k_s} waste4 + D_j$	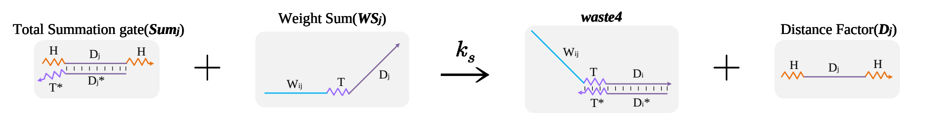

Figure S4: CRN model and reaction process of the absolute value summation module, where $k_s = 3 \times 10^5 M^{-1}s^{-1}$, $k_{rs} = 26s^{-1}$.

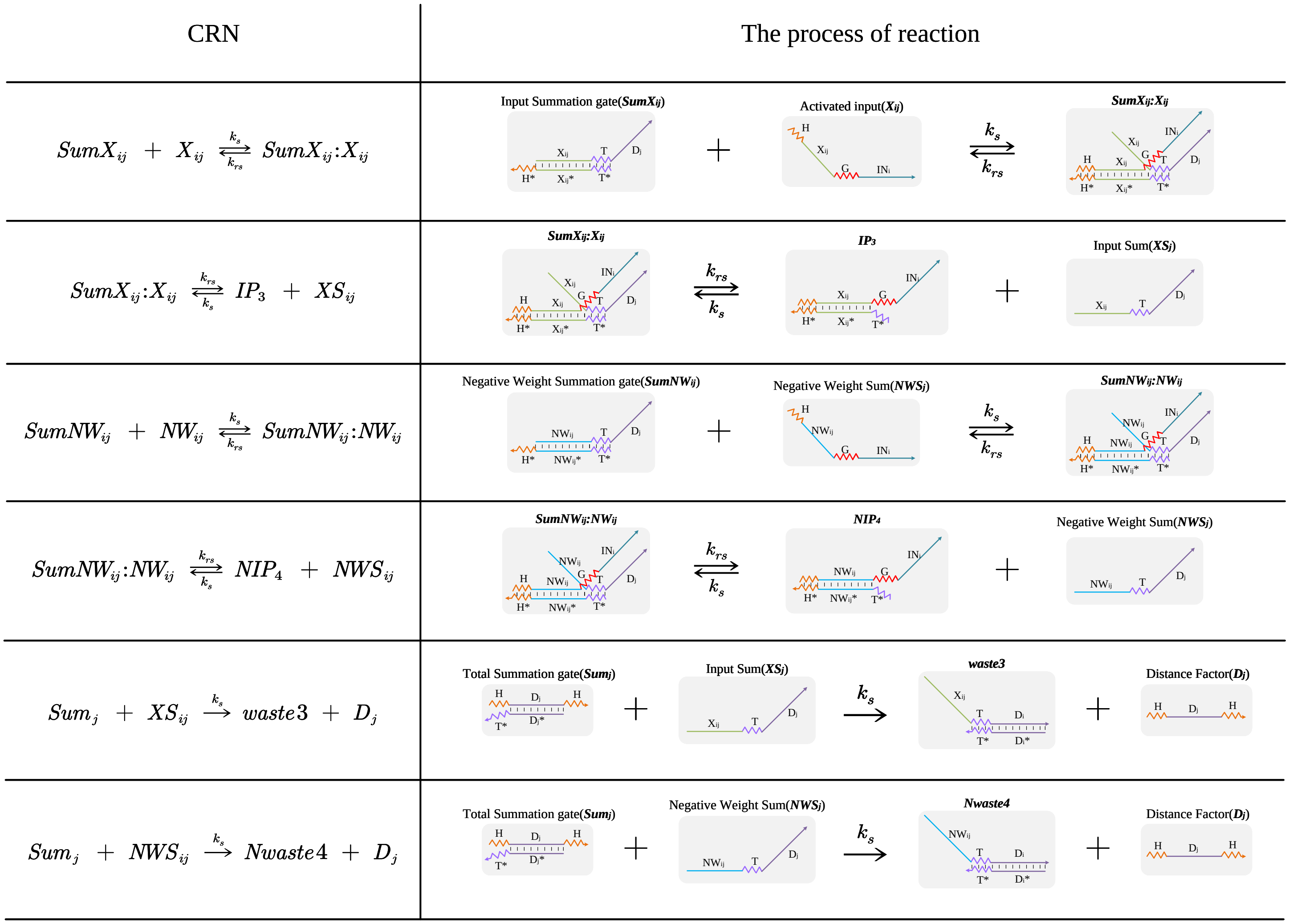

Figure S5: CRN model and reaction process of the absolute value summation module when the weight is negative, where $k_s = 3 \times 10^5 M^{-1}s^{-1}$, $k_{rs} = 26s^{-1}$.

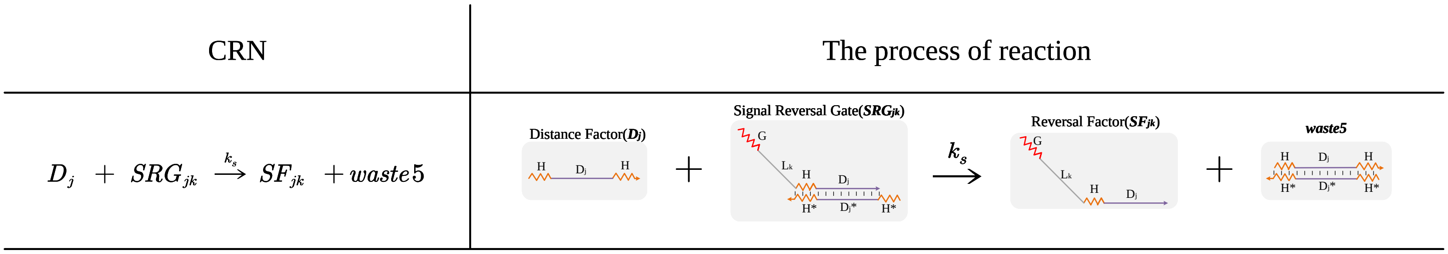

Figure S6: CRN model and reaction process of the signal reversal module, where $k_s = 3 \times 10^5 M^{-1}s^{-1}$.

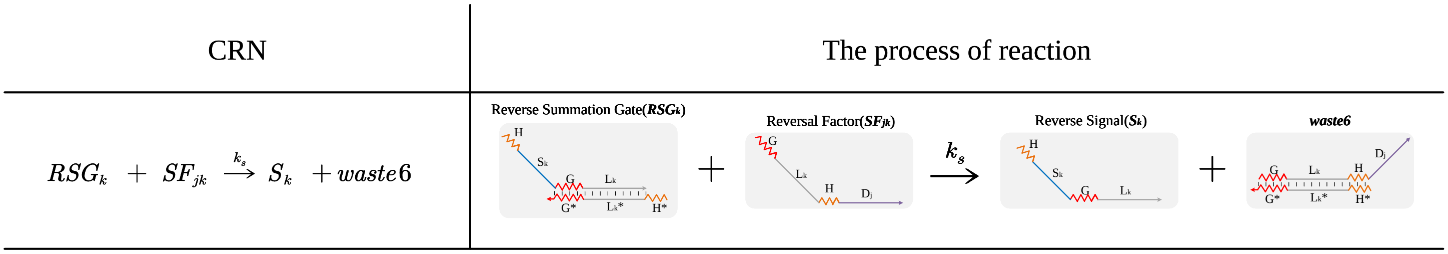

Figure S7: CRN model and reaction process of the reverse summation module, where $k_s = 3 \times 10^5 M^{-1}s^{-1}$.

CRN	The process of reaction
$Anh_{kj} + S_k \underset{k_{rf}}{\overset{k_f}{\rightleftharpoons}} Anh_{kj}{:}S_k$	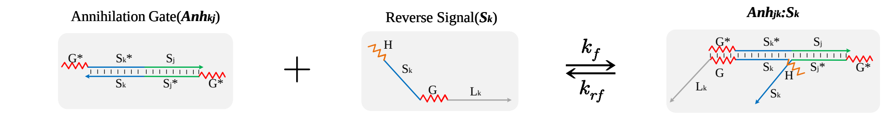
$Anh_{kj}{:}S_k + S_j \xrightarrow{k_f} waste7 + waste8$	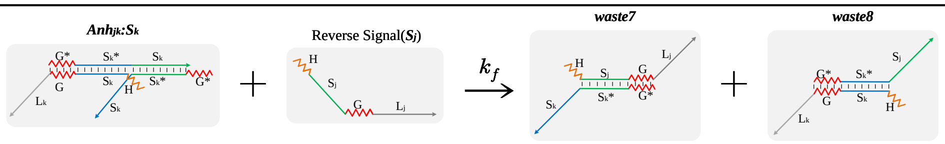
$Anh_{kj} + S_j \underset{k_{rf}}{\overset{k_f}{\rightleftharpoons}} Anh_{kj}{:}S_j$	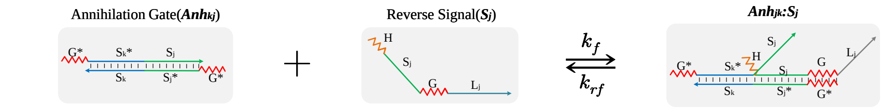
$Anh_{kj}{:}S_j + S_k \xrightarrow{k_f} waste7 + waste8$	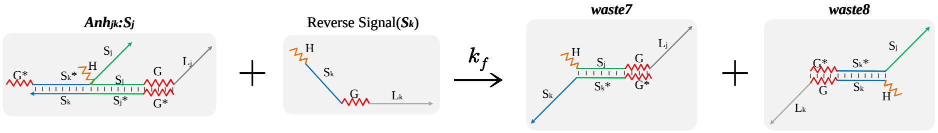

Figure S8: CRN model and reaction process of the annihilation module, where $k_f = 9 \times 10^7 M^{-1}s^{-1}$, $k_{rf} = 0.1s^{-1}$.

CRN	The process of reaction
$RE_{kl} + S_k \underset{k_{rs}}{\overset{k_s}{\rightleftharpoons}} RE_{kl}{:}S_k$	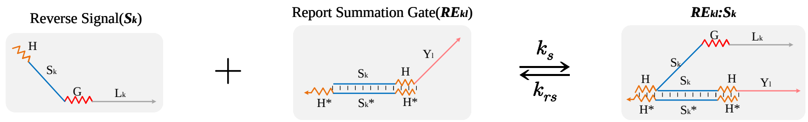
$RE_{kl}{:}S_k \underset{k_s}{\overset{k_{rs}}{\rightleftharpoons}} IP_5 + RY_{kl}$	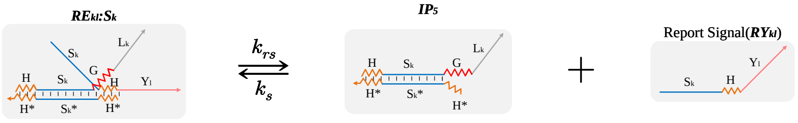

Figure S9: CRN model and reaction process of the report summation module., where $k_s = 3 \times 10^5 M^{-1}s^{-1}$, $k_{rs} = 26s^{-1}$.

CRN	The process of reaction
$D_j + SRG_{jk} \xrightarrow{k_s} SF_{jk} + waste5$	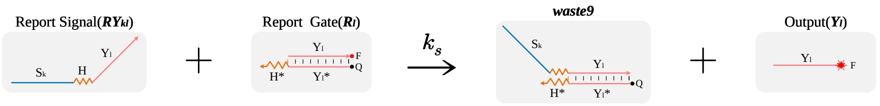

Figure S10: CRN model and reaction process of the reporting reaction module, where $k_s = 3 \times 10^5 M^{-1}s^{-1}$.

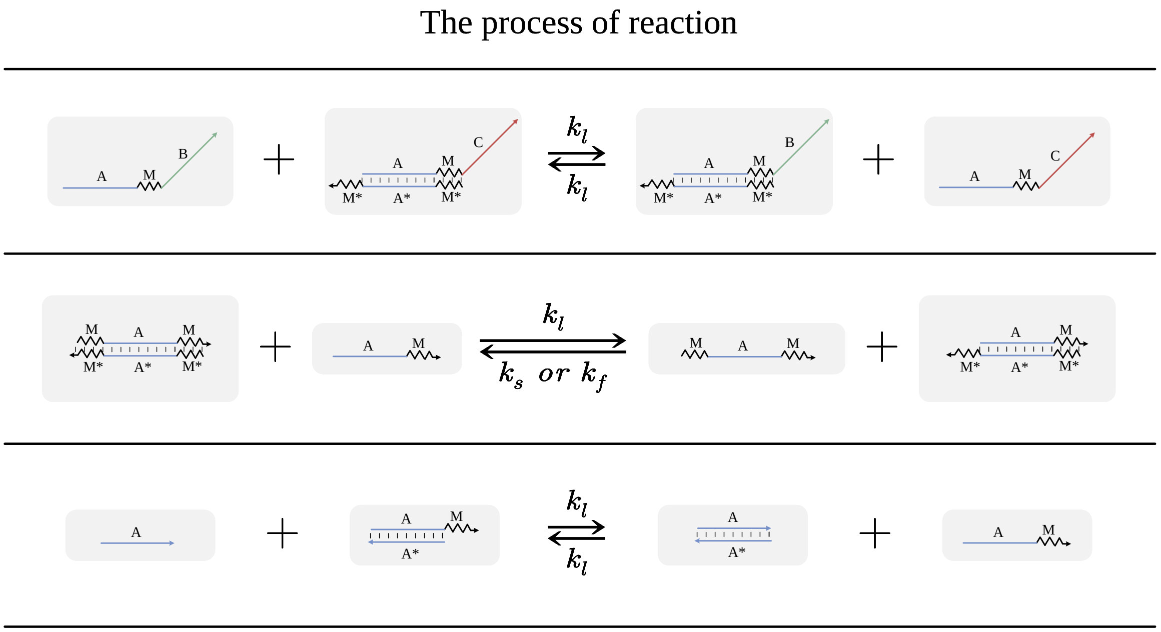

Figure S11: Possible leakage reaction processes ,where$k_l = 1M^{-1}s^{-1}$,$k_s = 3 \times 10^5 M^{-1}s^{-1}$, $k_f = 9 \times 10^7 M^{-1}s^{-1}$. Domains A, B, C represent arbitrary migration domains in branched structures, and M represents any small toehold. This figure illustrates three types of leakage reactions that may occur in the context of this paper. Additional arbitrary domains or toeholds may be attached to the 3' or 5' ends of each strand.

S2 Analysis of Leak Reactions

To validate the stability of the DNA-based Learning Vector Quantization (LVQ) neural network constructed in this paper, we conducted a leakage reaction analysis on each module of the network structure by introducing leakage reactions with varying rates: $0M^{-1}s^{-1}$, $1M^{-1}s^{-1}$, $1 \times 10^1 M^{-1}s^{-1}$, $1 \times 10^2 M^{-1}s^{-1}$, $1 \times 10^3 M^{-1}s^{-1}$, $1 \times 10^4 M^{-1}s^{-1}$, $1 \times 10^5 M^{-1}s^{-1}$. The concentrations of all species required for the reactions have been specified in the main text.

S2.1 Analysis of Leak Reactions in the Input Layer of DNA-Based LVQNN

The input layer of the DNA-based LVQNN is comprised of three modules: the input activation module, the subtraction annihilation module, and the absolute value summation module. The functionality of the input layer is realized through a Manhattan distance solving model. The testing is conducted under two scenarios: when the weight values are positive and when the weight values are negative. Due to the numerical span of the input data ranging from 0 to 1000nM, three concentration levels are selected for each scenario: 0-10nM, 10-100nM, and 1000-1000nM (note: the last range should likely be a typo and meant to be 100-1000nM). For each concentration level, representative data points are selected for examination, including three cases: when the concentration of the input X is equal to the concentration W of, when the concentration of the input X is less than the concentration of W, and when the concentration of the input X is greater than the concentration of W.

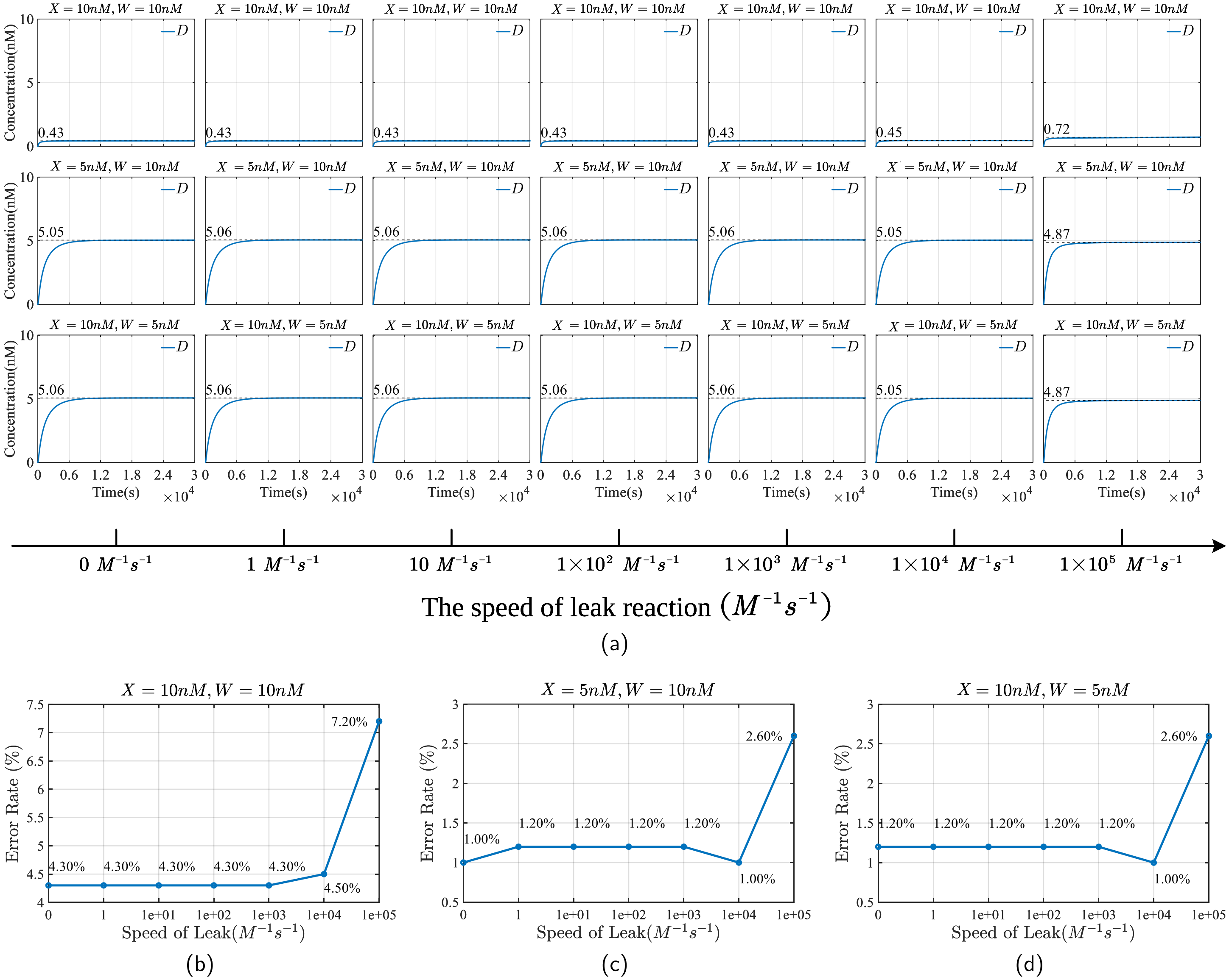

Figure S12: Leakage reaction analysis at 0-10nM concentration level when weights are positive. (a). Output results under different leakage reactions. (b). Variation of error rate when input equals weight. (c). Variation of error rate when input is less than weight. (d). Variation of error rate when input is greater than weight.

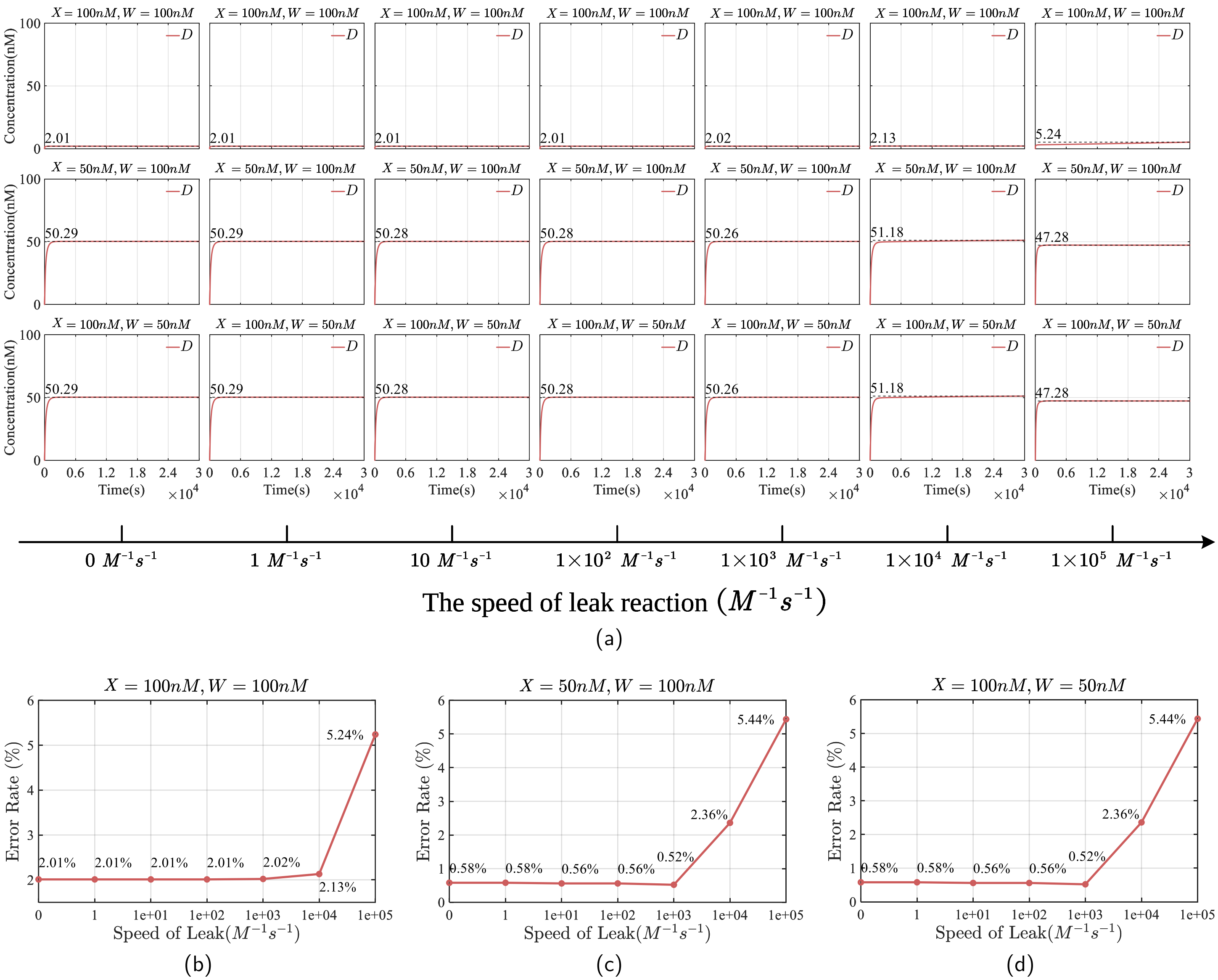

Figure S13: Leakage reaction analysis at 10-100nM concentration level when weights are positive. (a). Output results under different leakage reactions. (b). Variation of error rate when input equals weight. (c). Variation of error rate when input is less than weight. (d). Variation of error rate when input is greater than weight.

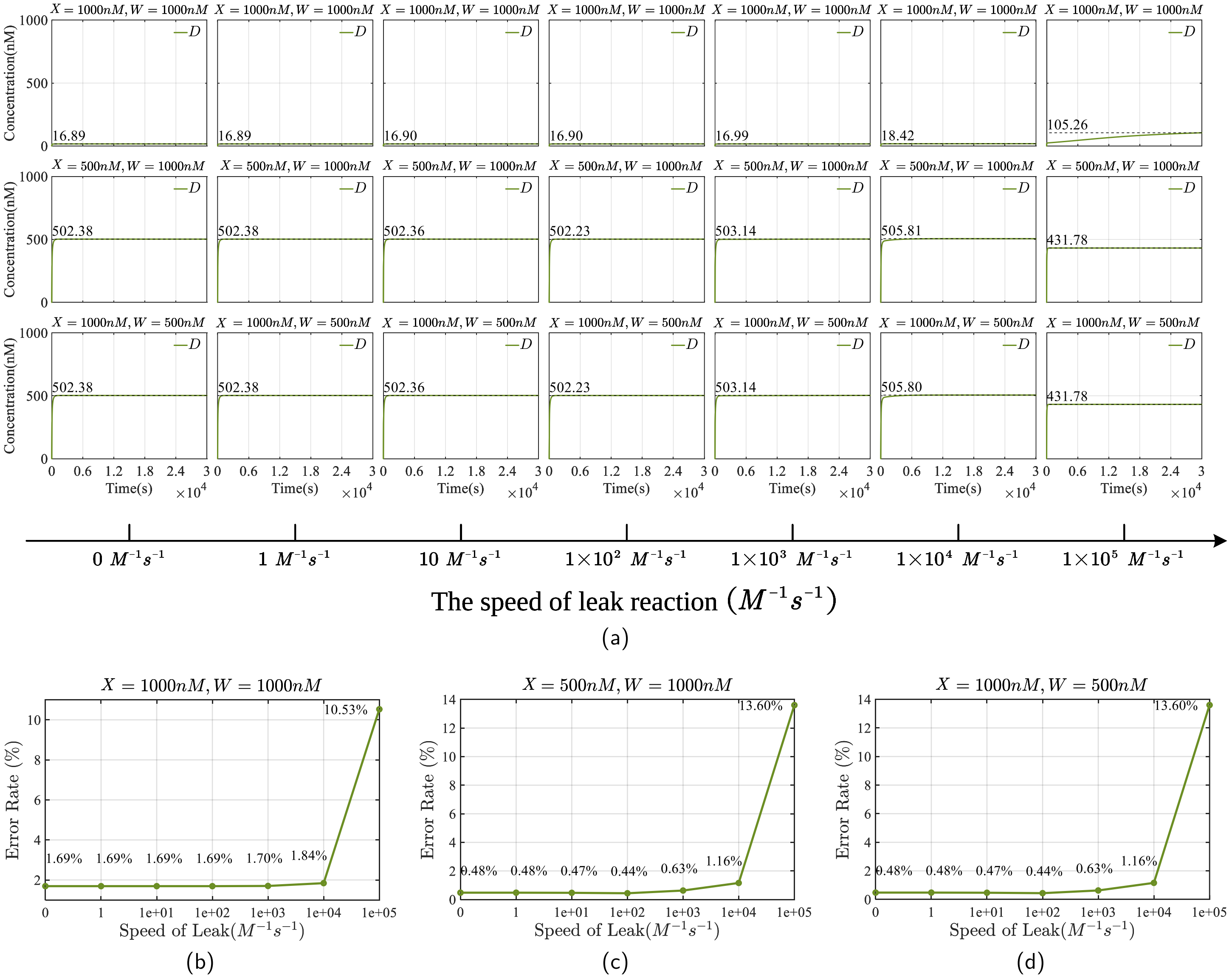

Figure S14: Leakage reaction analysis at 100-1000nM concentration level when weights are positive. (a). Output results under different leakage reactions. (b). Variation of error rate when input equals weight. (c). Variation of error rate when input is less than weight. (d). Variation of error rate when input is greater than weight.

As shown in Figure S12(a), the output results under different leakage reaction rates within the 0-10nM concentration level are presented. Figures S12(b), S12(c), and S12(d) indicate that when the leakage reaction rate is within the range of $0 - 1 \times 10^4 M^{-1}s^{-1}$, there is little to no change in the error rate of the output results. It is only when the leakage reaction rate reaches an astonishing $1 \times 10^5 M^{-1}s^{-1}$ that a significant increase in the error rate of the output results occurs. Next, we analyze the stability of the Euclidean distance solving model within the 10-100nM range. As shown in Figure S13(a), the output results under different leakage reaction rates within the 10-100nM concentration level are presented. Figures S13(b), S13(c), and S13(d) show that when the leakage reaction rate is within the range of $0 - 1 \times 10^4 M^{-1}s^{-1}$, there is little to no change in the error rate of the output results. It is only when the leakage reaction rate reaches $1 \times 10^5 M^{-1}s^{-1}$ that a significant increase in the error rate of the output results is observed. Similarly, Figure S14(a) displays the output results under different leakage reaction rates within the 100-1000nM range. Figures S14(b), S14(c), and S14(d) indicate that when the leakage reaction rate is within the range of $0 - 1 \times 10^4 M^{-1}s^{-1}$, there is little to no change in the error rate of the output results, and a significant increase in the error rate occurs only when the leakage reaction rate reaches $1 \times 10^5 M^{-1}s^{-1}$. In summary, when the weights are positive, a significant increase in the error rate is observed only when the leakage reaction rate reaches an astonishing $1 \times 10^4 M^{-1}s^{-1}$. Next, we analyze

the impact of leakage reactions with different speeds on the output results when the weights are negative.

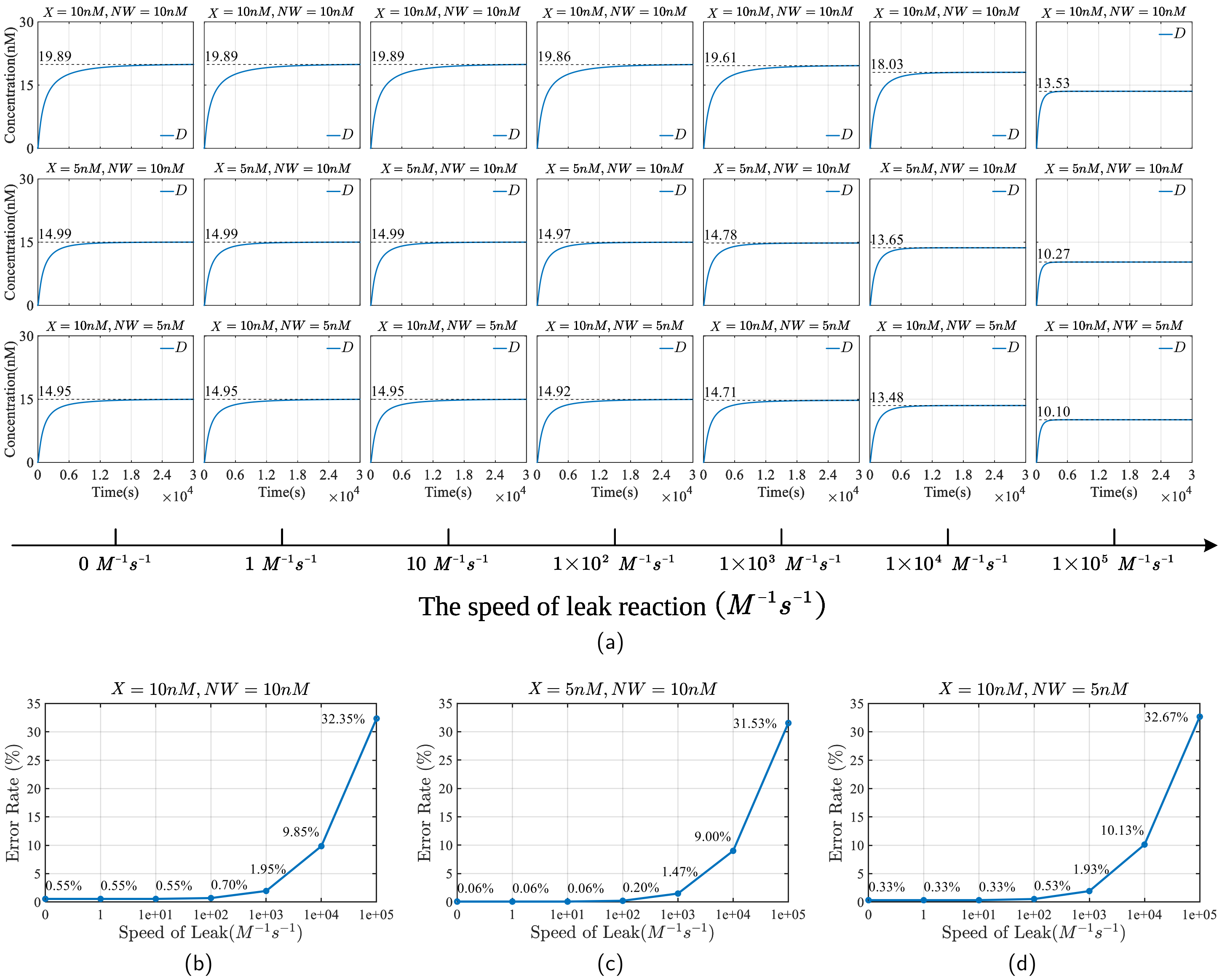

Figure S15: Leakage reaction analysis at 0-10nM concentration level when weights are negative. (a). Output results of different leakage reaction. (b). Variation of error rate when input equals negative weight. (c). Variation of error rate when input is less than negative weight. (d). Variation of error rate when input is greater than negative weight.

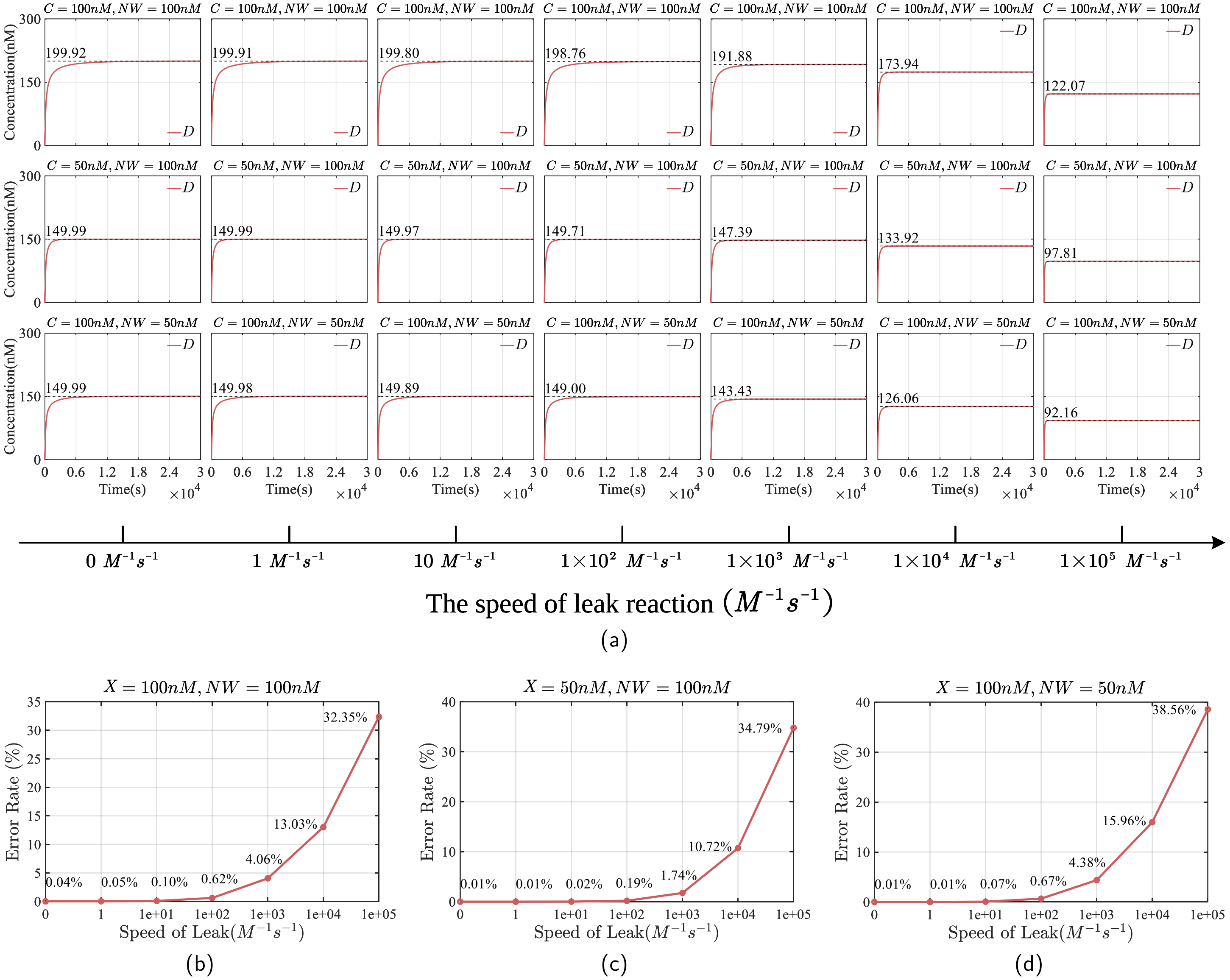

Figure S16: Leakage reaction analysis at 10-100nM concentration level when weights are positive. (a). Output results of different leakage reaction. (b). Variation of error rate when input equals negative weight. (c). Variation of error rate when input is less than negative weight. (d). Variation of error rate when input is greater than negative weight.

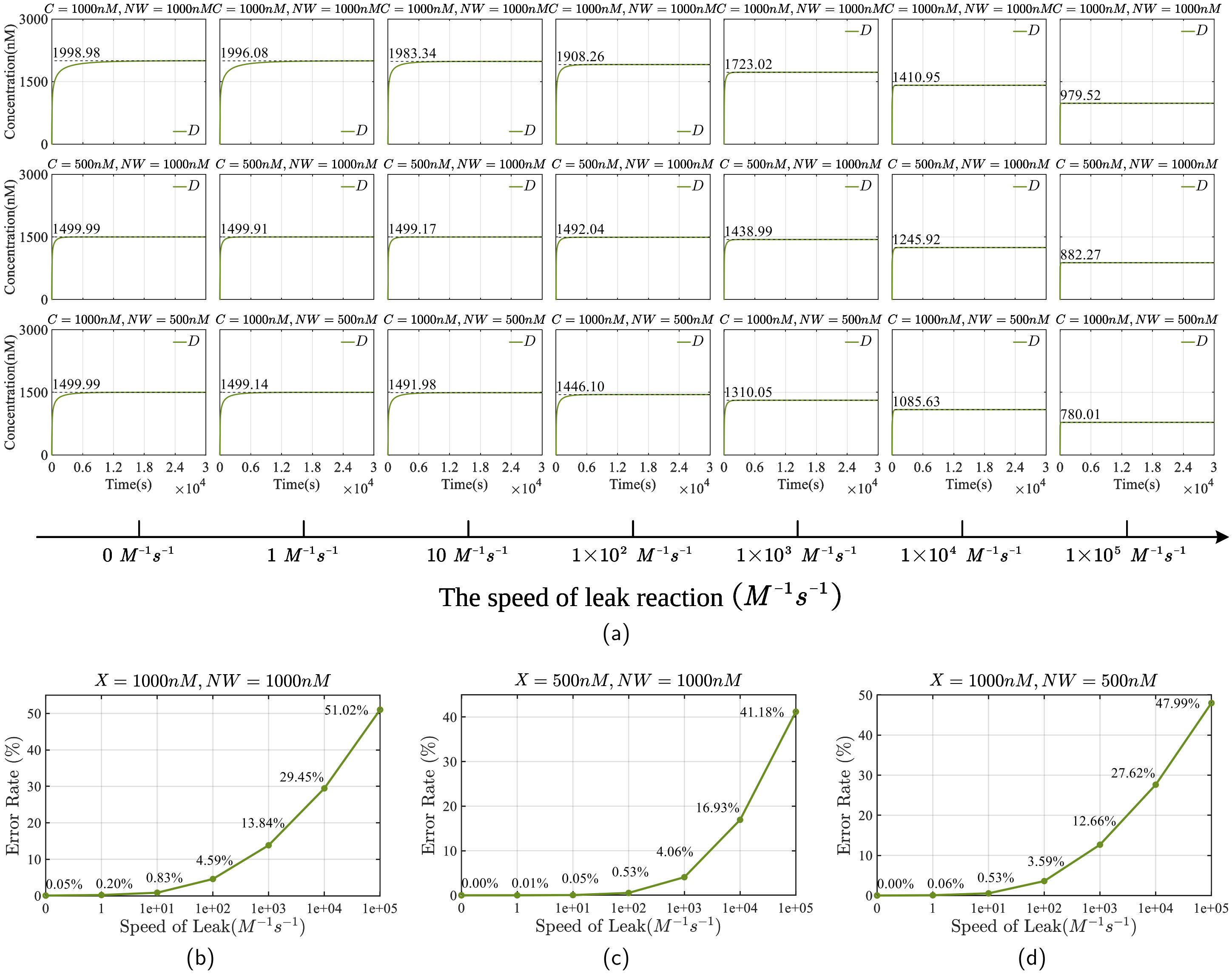

Figure S17: Leakage reaction analysis at 100-1000nM concentration level when weights are positive. (a). Output results of different leakage reaction. (b). Variation of error rate when input equals negative weight. (c). Variation of error rate when input is less than negative weight. (d). Variation of error rate when input is greater than negative weight.

Next, we analyze the impact of leakage reaction speeds on the output results under different conditions when the weights are negative. As shown in Figure S15(a), the output results under various leakage reaction speeds within the 0-10nM range with negative weights are presented. Figures S15(b), S15(c), and S15(d) illustrate that when the leakage reaction speed falls within the range of $0 - 1 \times 10^3 M^{-1}s^{-1}$, the variation in error rate of the output results is minimal or non-existent, and the error rate remains below 2%. Only when the leakage reaction speed reaches $1 \times 10^4 M^{-1}s^{-1}$ does the error rate undergo a significant increase. Similarly, in Figure S16(a), the output results under various leakage reaction speeds within the 10-100nM range with negative weights are depicted. Figures S16(b), S16(c), and S16(d) show that within the range of $0 - 1 \times 10^3 M^{-1}s^{-1}$, the error rate variation remains small or non-existent, with an error rate less than 2%, and a significant increase in error rate occurs only when the leakage reaction speed reaches $1 \times 10^4 M^{-1}s^{-1}$. Furthermore, Figure S17(a) presents the output results under different leakage reaction speeds within the 0-10nM range with negative weights. Figures S17(b), S17(c), and S17(d) indicate that When the leakage response speed falls within the range of $0 - 1 \times 10^2 M^{-1}s^{-1}$, the error rate variation is minimal or non-existent. However, a notable increase in error rate occurs when the leakage reaction speed reaches $1 \times 10^3 M^{-1}s^{-1}$, which may be attributed to the larger concentration range making leakage reactions more prone to occur. In summary, when the weights are negative, a significant increase in error

rate occurs only when the leakage reaction speed reaches a remarkable threshold of $1 \times 10^3 M^{-1}s^{-1}$ or higher.

The fundamental reactions in this paper are based on Qian's seesaw gate and annihilation gate, where the leakage reaction rate for Qian's seesaw gate is merely $1M^{-1}s^{-1}$[1]. However, the Manhattan distance solution model we constructed exhibits significant changes in its output results only when the leakage reaction reaches an astonishing $1 \times 10^4 M^{-1}s^{-1}$. This serves as ample evidence that the input activation module, subtraction annihilation module, and absolute value summation module constructed in this paper possess sufficient stability.

S2.2 Analysis of Leak Reactions in the Competitive and Output Layers of DNA-Based LVQNN

The DNA-based LVQNN competitive layer comprises three modules: a signal reversal module, a reverse summation module, and an annihilation module. The output layer, on the other hand, consists of two modules: a report summation module, and a reporting reaction module. The functionality of both the competitive layer and the output layer is realized through a winner-take-all network. As illustrated in Figure S18, different leakage rates are introduced into eight combinations of input scenarios for the winner-takes-all model. From the figure, it can be observed that when the leakage reaction rate falls within the range of $0 - 1 \times 10^3 M^{-1}s^{-1}$, there is no significant change in the output results. When the rate reaches $1 \times 10^4 M^{-1}s^{-1}$, the output concentration decreases by approximately 20%, but the accuracy of the results remains unchanged. Notably, even when the leakage reaction rate reaches an astonishing $1 \times 10^5 M^{-1}s^{-1}$, the output concentration decreases by approximately 60%, yet the accuracy of the results still remains constant. This demonstrates that the DNA-based LVQNN competitive layer and output layer possess excellent stability, capable of overcoming the effects of leakage reactions within the speed range of $1 \times 10^4 M^{-1}s^{-1}$.

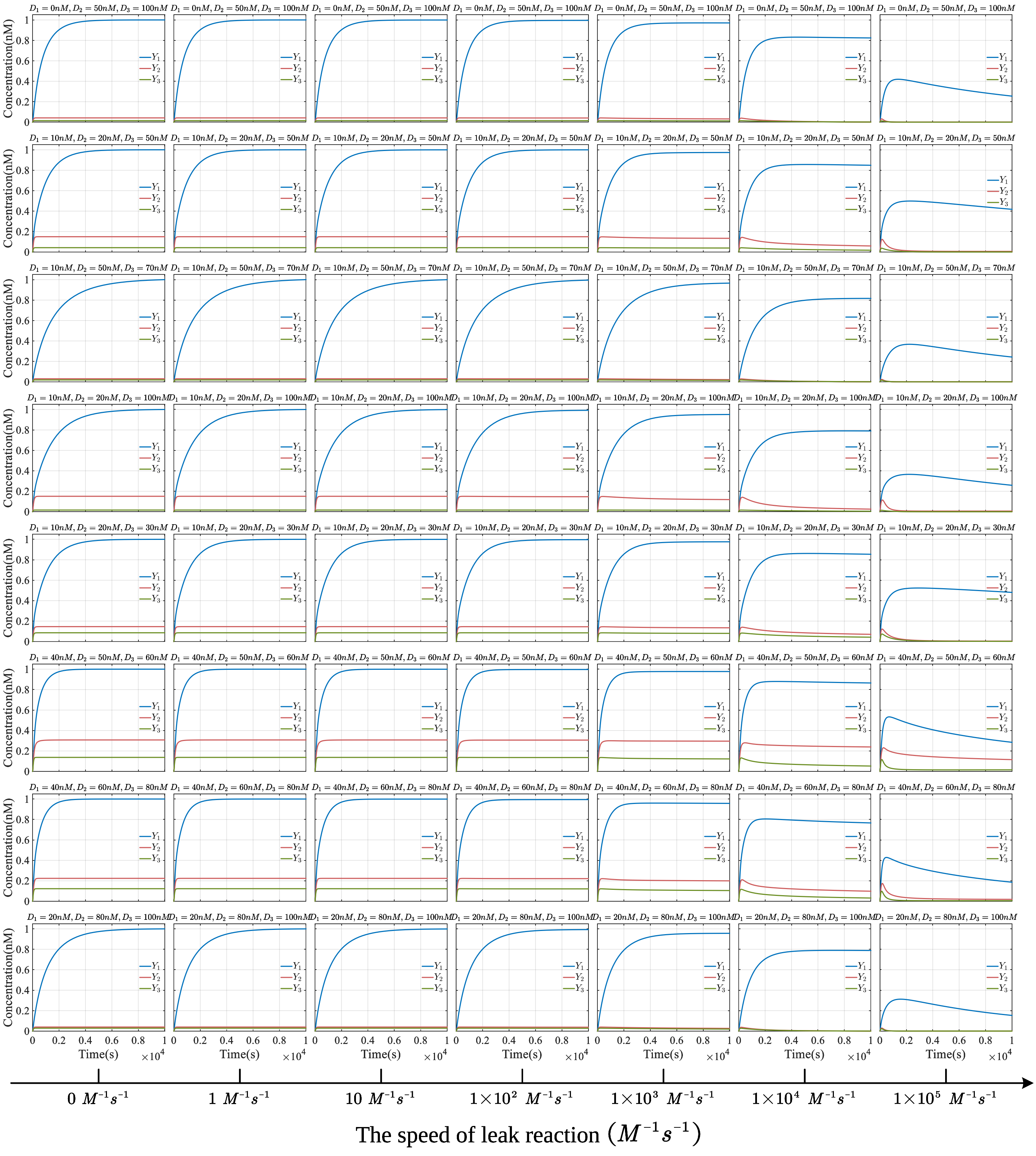

Figure S18: Output results of the loser-take-all network model under different leakage rates. The output results are normalized when the leakage reaction rate is 0.

S2.3 Analysis of Leak Reactions in the overall of DNA-Based LVQNN

Select 10 groups of breast tumor cases for DNA based LVQNN leakage analysis. Among them, there are five groups of benign cases and five groups of malignant cases. As shown in Figure S19, the diagnostic results of benign cases under different leakage speeds are presented. As shown in Figure S20, the diagnostic

results of benign cases under different leakage speeds are presented. From Figure S19, it can be seen that when the leakage reaction rate is between $0 - 1 \times 10^4 M^{-1}s^{-1}$, there is no significant change in the output results. When the rate reaches an astonishing $1 \times 10^5 M^{-1}s^{-1}$, the concentration of the output results decreases, but the accuracy of the results remains unchanged. The diagnostic results of the malignant case shown in Figure S20 have almost the same changes as Figure S19.

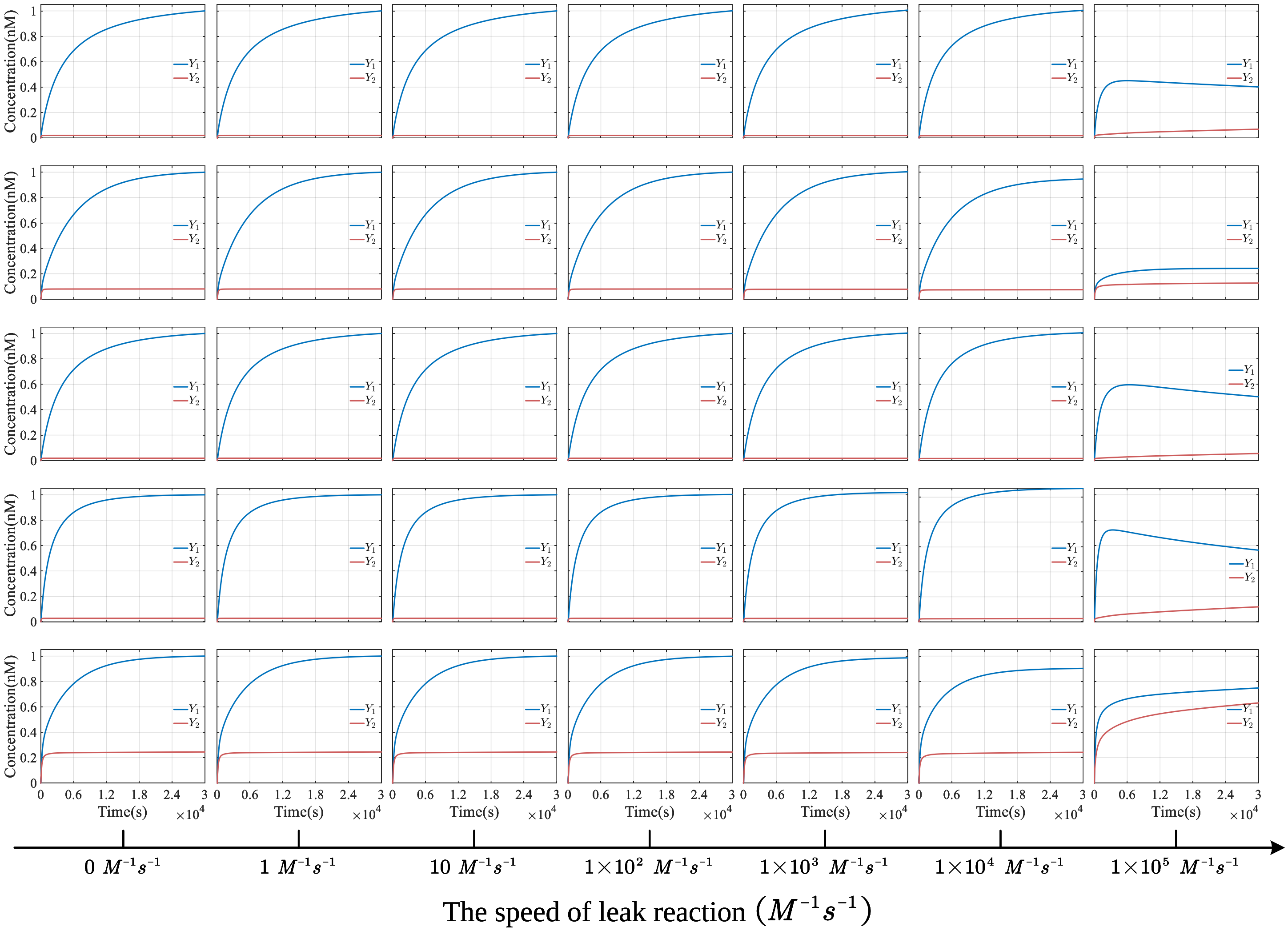

Figure S19: Diagnostic results of benign cases at different leakage rates. The output results are normalized separately when the leakage reaction rate is 0.

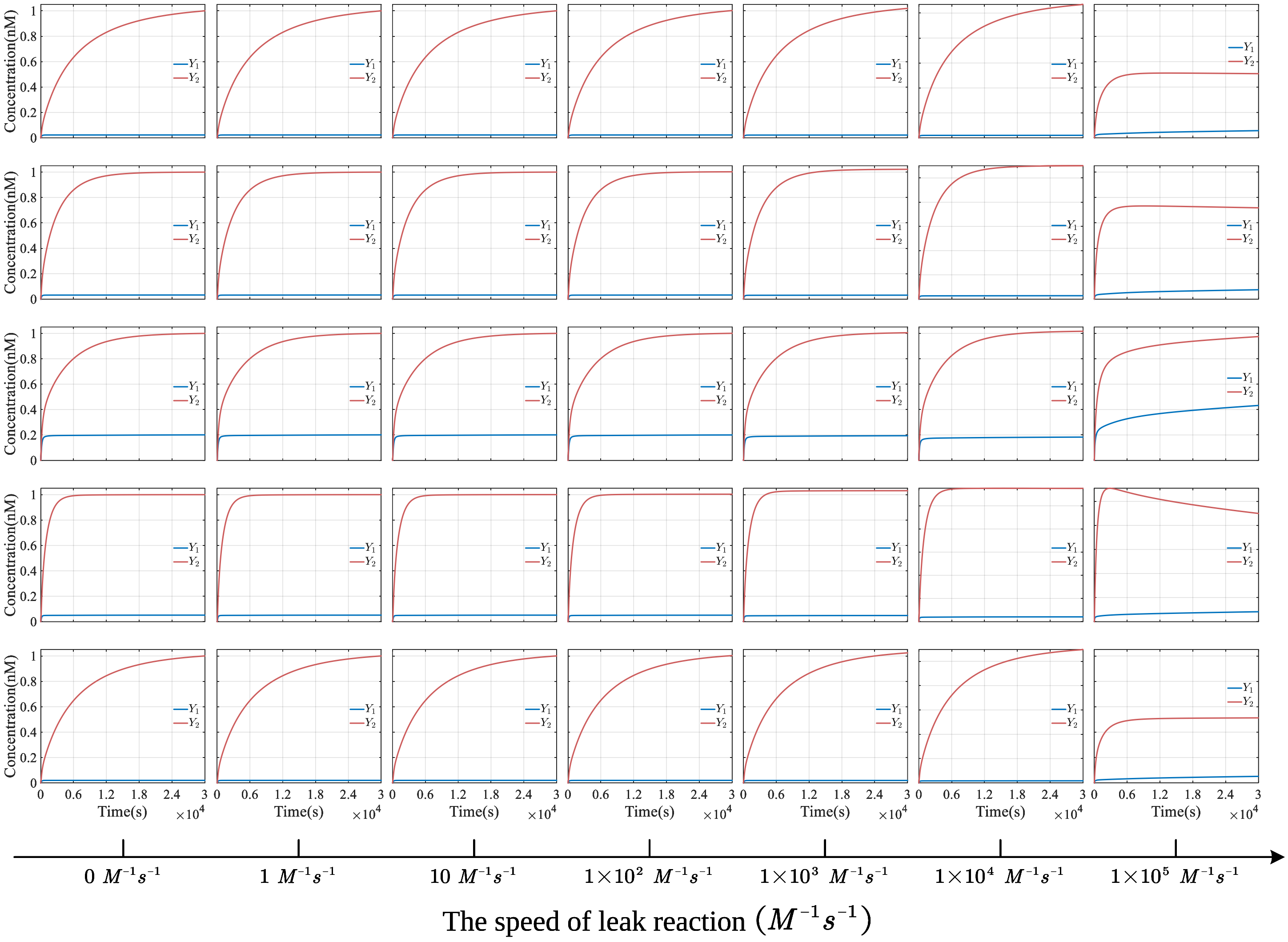

Figure S20: Diagnostic results of malignant cases at different leakage rates. The output results are normalized separately when the leakage reaction rate is 0.

In summary, the DNA based LVQNN constructed in this study exhibits excellent stability and can effectively resist the error effects caused by leakage reactions within the speed range of $1 \times 10^4 M^{-1}s^{-1}$. Although the resistance to leakage reactions varies among different modules, and the concentration of products generated by each module reaction mostly decreases with the increase of leakage reaction rate. However, the impact caused by the leakage reaction is proportional and equal, which will result in little change in the accuracy of the overall network operation results, only a decrease in the concentration of the output results. In order to address the issue of concentration reduction in actual reactions, signal recovery gates may be added in subsequent experiments to maintain output stability.

S3 Complete Reaction Concentration Curves

This section displays the complete output concentration curves for the two diagnostic modes discussed in the main text.

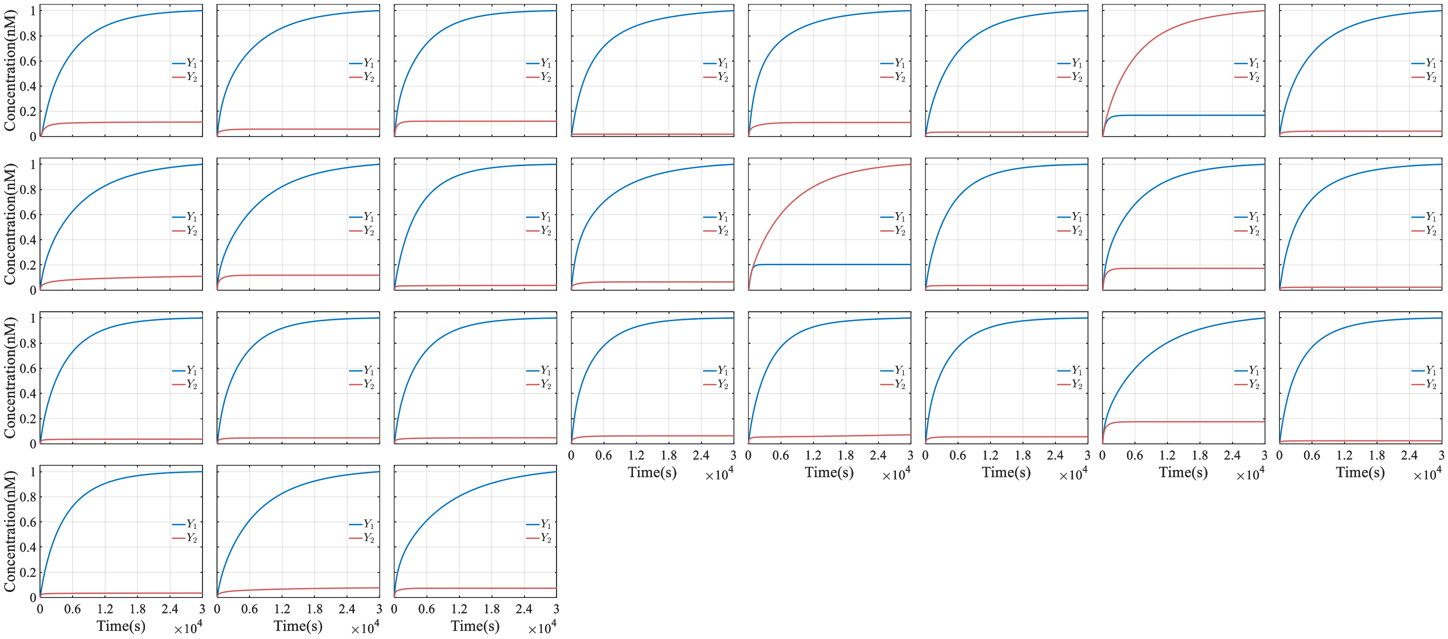

Figure S21: Complete output concentration curves corresponding to Figure 5(b) in the main text, where Y_1 represents a benign tumor diagnosis and Y_2 represents a malignant tumor diagnosis.

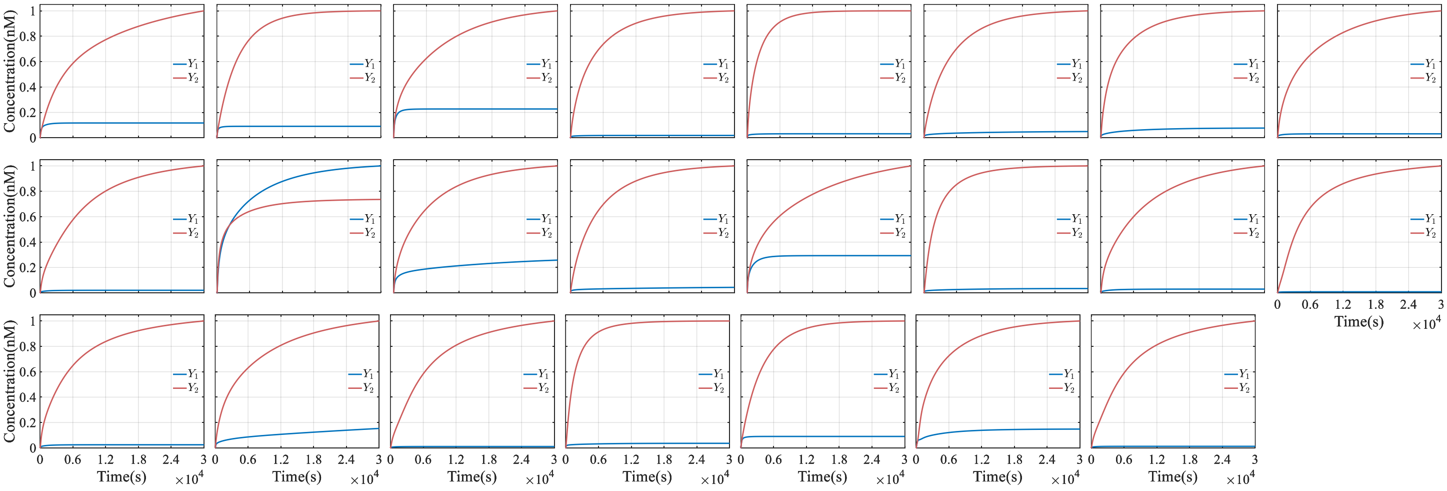

Figure S22: Complete output concentration curves corresponding to Figure 5(c) in the main text, where Y_1 represents a benign tumor diagnosis and Y_2 represents a malignant tumor diagnosis.

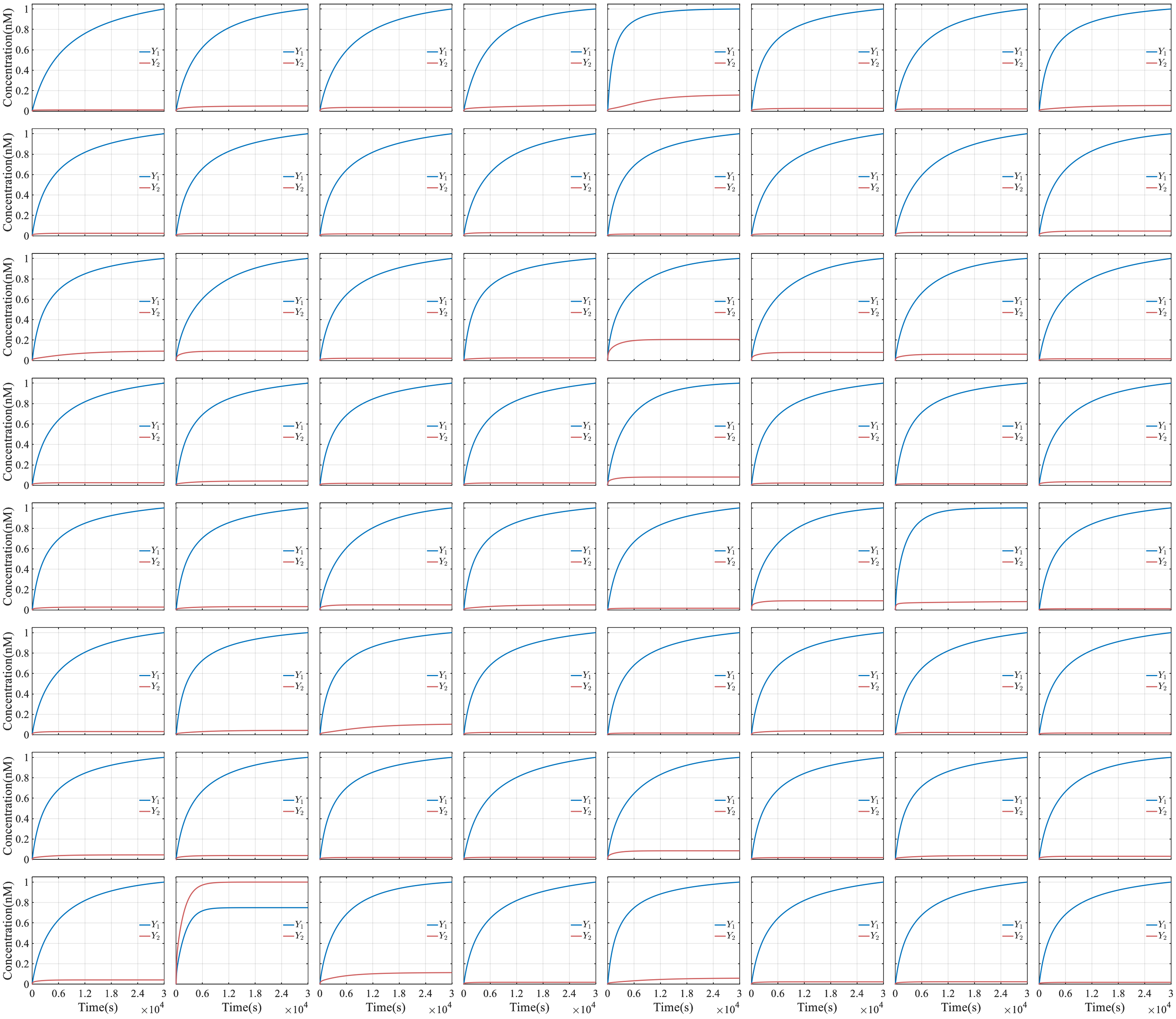

Figure S23: Complete output concentration curves corresponding to Figure 6(c) in the main text, where Y_1 represents a diagnosis of BRCA and Y_2 represents a healthy diagnosis.

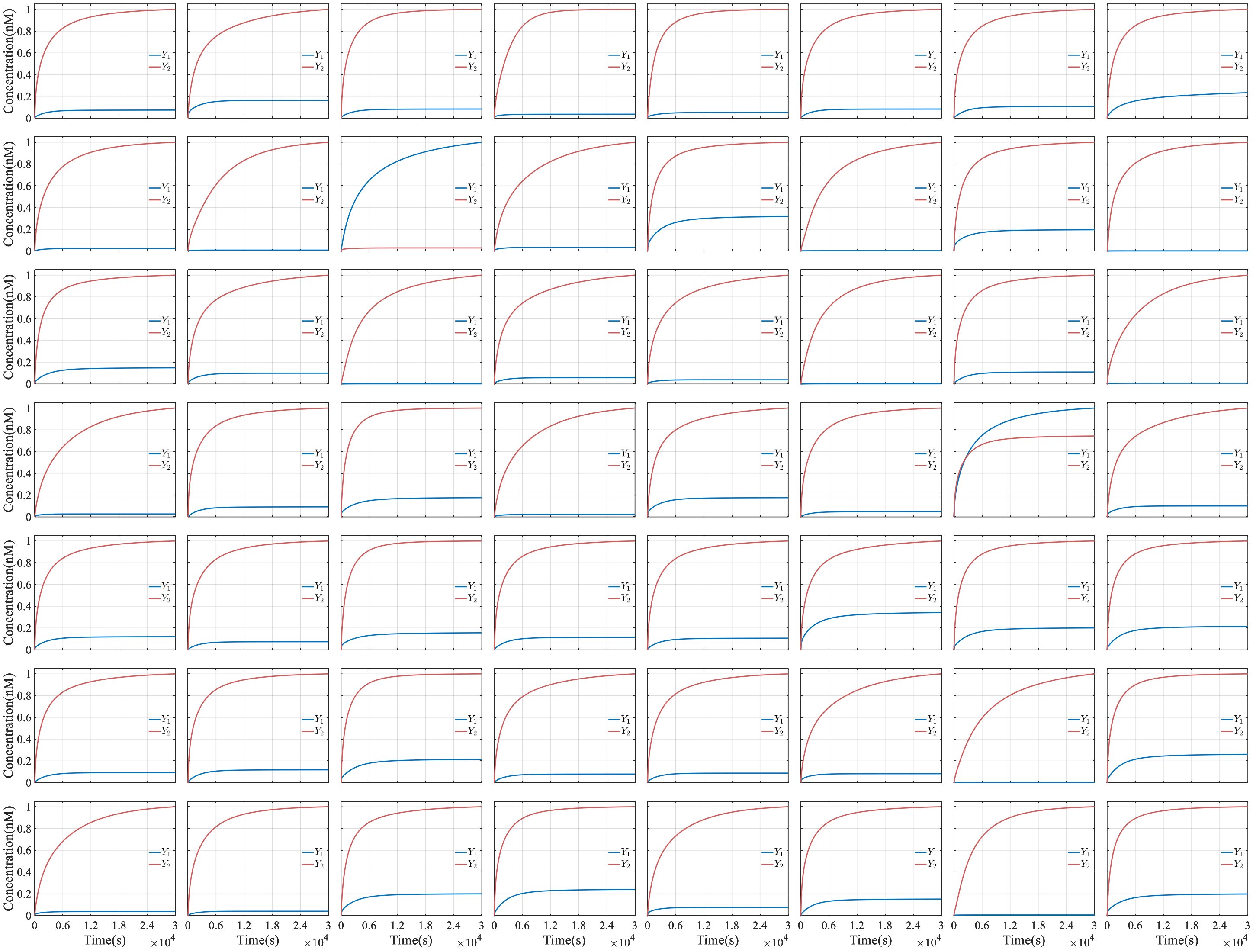

Figure S24: Complete output concentration curves corresponding to Figure 6(e) in the main text, where Y_1 represents a diagnosis of BRCA and Y_2 represents a healthy diagnosis.

References

[1] L. Qian, E. Winfree, *SCIENCE* **2011**, *332*, 6034 1196.
